# VO_x_‐Based Non‐Volatile Radio‐Frequency Switches for Reconfigurable Filter

**DOI:** 10.1002/advs.202501989

**Published:** 2025-05-28

**Authors:** Dabin Seo, Dahyeon Kim, Jiyeon Ryu, Changwoo Pyo, Seungchan Lee, Tae‐Sik Yoon, Myungsoo Kim

**Affiliations:** ^1^ Graduate School of Semiconductor Materials and Devices Engineering Ulsan National Institute of Science and Technology (UNIST) Ulsan 44919 South Korea; ^2^ Department of Electrical Engineering Ulsan National Institute of Science and Technology (UNIST) Ulsan 44919 South Korea; ^3^ Department of Materials Science and Engineering Ulsan National Institute of Science and Technology (UNIST) Ulsan 44919 South Korea

**Keywords:** 5G/6G communication, non‐volatile memory, radio‐frequency switch, reconfigurable filter, vanadium oxide

## Abstract

Vanadium oxide (VO_x_) based memristor is a promising candidate for next‐generation non‐volatile memory and radio‐frequency (RF) switches due to its compatibility with wafer‐level integration and high‐frequency operation. This work demonstrates high‐performance VO_x_ memristor with gold and silver electrodes, achieving a higher cutoff frequency compared to previously reported VO_x_ devices. The devices exhibit long retention, high endurance (≈10^3^ cycles), and nanosecond switching speeds, enabling the fabrication of RF switches with a cutoff frequency of ≈4.5 THz, low insertion loss (< 0.46 dB), and high isolation (>20 dB) from 0.1 to 20 GHz with stable operation extended to frequency up to 67 GHz. Leveraging these switches, a reconfigurable X‐band bandpass filter whose is realized center frequency is tuned from 8.2 GHz in the OFF state to 7.6 GHz in the ON state, achieving a tunable range of ≈600 MHz. This demonstration paves the way for compact and versatile RF front‐ends with improved frequency agility in advanced communication systems.

## Graphical abstract

Vanadium oxide‐based memristor devices achieve high performance for next‐generation non‐volatile memory and RF switches. These devices demonstrate a cutoff frequency of ≈4.5 THz, low insertion loss (<0.46 dB), and high isolation (>20 dB) from 0.1 to 20 GHz with stable operation extended to 67 GHz. Leveraging these switches, a reconfigurable X‐band bandpass filter is realized for advanced RF systems.

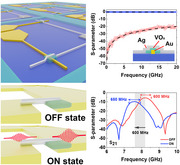

## 1 Introduction

The burgeoning growth of wireless communication technologies, spurred by advancements in 5G/6G, the Internet of Things, and autonomous systems, drives an unprecedented demand for reconfigurable radio‐frequency (RF) front‐end systems.^[^ ^1^, ^2^, ^3^, ^4^, ^5^ ^]^ To dynamically adapt to diverse operating frequency, bandwidths, and communication standards, these systems require components that offer both flexibility and efficiency, with the RF switch central to their operation as a critical element enabling rapid and efficient signal routing between multiple circuit paths.^[^ ^6^, ^7^, ^8^, ^9^, ^10^, ^11^ ^]^ While traditionally dominated by semiconductor technologies like p‐type intrinsic n‐type (PIN) diodes and field‐effect transistors, conventional RF switches present limitations, including high insertion loss, degraded isolation at higher frequency, and most significantly, the requirement of continuous power consumption to maintain their operational state (static power consumption), even when no signal is being transmitted.^[^ ^12^, ^13^, ^14^ ^]^ These limitations pose substantial challenges for battery‐powered devices and energy‐conscious applications.

To overcome these drawbacks, memristive devices such as resistive random‐access memory (ReRAM), conductive‐bridging RAM (CBRAM), and phase‐change memory (PCM) have recently gained considerable attention as promising candidates for implementing next‐generation RF switches.^[^ ^15^, ^16^, ^17^ ^]^ Memristive devices exploit a nonvolatile resistive switching mechanism, whereby the resistance of the device is reversibly modulated between a high resistance state (HRS) and a low resistance state (LRS) in response to an applied voltage or current.^[^ ^18^, ^19^ ^]^ This switching is typically associated with the formation and rupture of conductive pathways, often referred to as filaments, driven by electrochemical redox reactions, the migration of mobile ions, or valence change processes that locally alter the material's conductivity.^[^ ^20^, ^21^ ^]^ This inherent non‐volatility eliminates static power consumption, offering a decisive advantage over traditional volatile switches. The potential of memristor for achieving low insertion loss and high isolation across a wide frequency range has been demonstrated in several recent studies, exploring materials such as TaO_x_, HfO_x_, TiO_x_.^[^ ^22^, ^23^, ^24^ ^]^ Particularly, structural engineering approaches like multi‐material stacks further enhance their durability and pave the way for the development of highly reliable memristive RF switches. Despite these advancements, challenges persist regarding switching speed, endurance, and seamless integration with existing RF circuit architectures.

Among various memristor materials, vanadium oxide (VO_x_), a transition metal oxide, has attracted considerable attention due to its distinctive material properties. Vanadium's rich polymorphism, characterized by multiple oxidation states (e.g., V^2+^, V^3+^, V^4+^, V^5+^) and the formation of numerous stable and metastable phases, including the Magnéli phases (V_n_O_2n−1_), provides a versatile platform for tailoring electrical and structural properties for various electronic applications. In addition, mobile dopants such as interstitial boron within VO_x_ exhibit high diffusivity, imparting intrinsic metallic properties to the material.^[^ ^25^, ^26^ ^]^ This behavior indicates that VO_x_ is capable of accommodating metallic ions with high mobility, allowing them to migrate efficiently within its layers. These unique characteristics make VO_x_ a promising material in memristor applications, where it has demonstrated desirable attributes such as rapid switching speeds, robust endurance, and scalability for nanoscale device integration.^[^ ^27^, ^28^, ^29^, ^30^, ^31^ ^]^

This study leverages the distinctive properties of VO_x_ to develop high‐performance memristive devices employing gold (Au) and silver (Ag) electrodes, forming conductive filaments, achieving a significantly improved figure of merit compared to previously reported oxide‐based devices. Our devices represent exceptional characteristics, including extended retention (> 60 h), high endurance (≈10^3^ cycles), and nanosecond‐scale switching speeds. Building upon these Ag/VO_x_/Au devices, we successfully fabricated RF switches that exhibit a cutoff frequency of ≈4.5 THz, low insertion loss (< 0.46 dB), and high isolation (> 20 dB) across a frequency range of 0.1 to 20 GHz, while maintaining stable operation across an extended frequency range up to 67 GHz. To demonstrate practical applicability, a reconfigurable X‐band bandpass filter was implemented using these switches, achieving a tunable center frequency (7.6–8.2 GHz). In the OFF state, the filter exhibits a 3‐dB bandwidth of ≈600 MHz with an insertion loss of 5.6 dB, while in the ON state, it achieves a 3‐dB bandwidth of ≈650 MHz with an insertion loss of 9.6 dB. The filter also maintains a good return loss (> 20.2 dB), along with additional advantages of fast switching speeds, and zero‐static power dissipation due to their non‐volatile nature. These findings highlight the substantial potential of VO_x_‐based memristor technology to enable the creation of compact, versatile, and frequency‐agile RF front‐ends for the next‐generation of advanced communication systems.

## 2 Results and Discussion

### 2.1 Device Schematics and Images with Material Characterization

**Figure 1:**
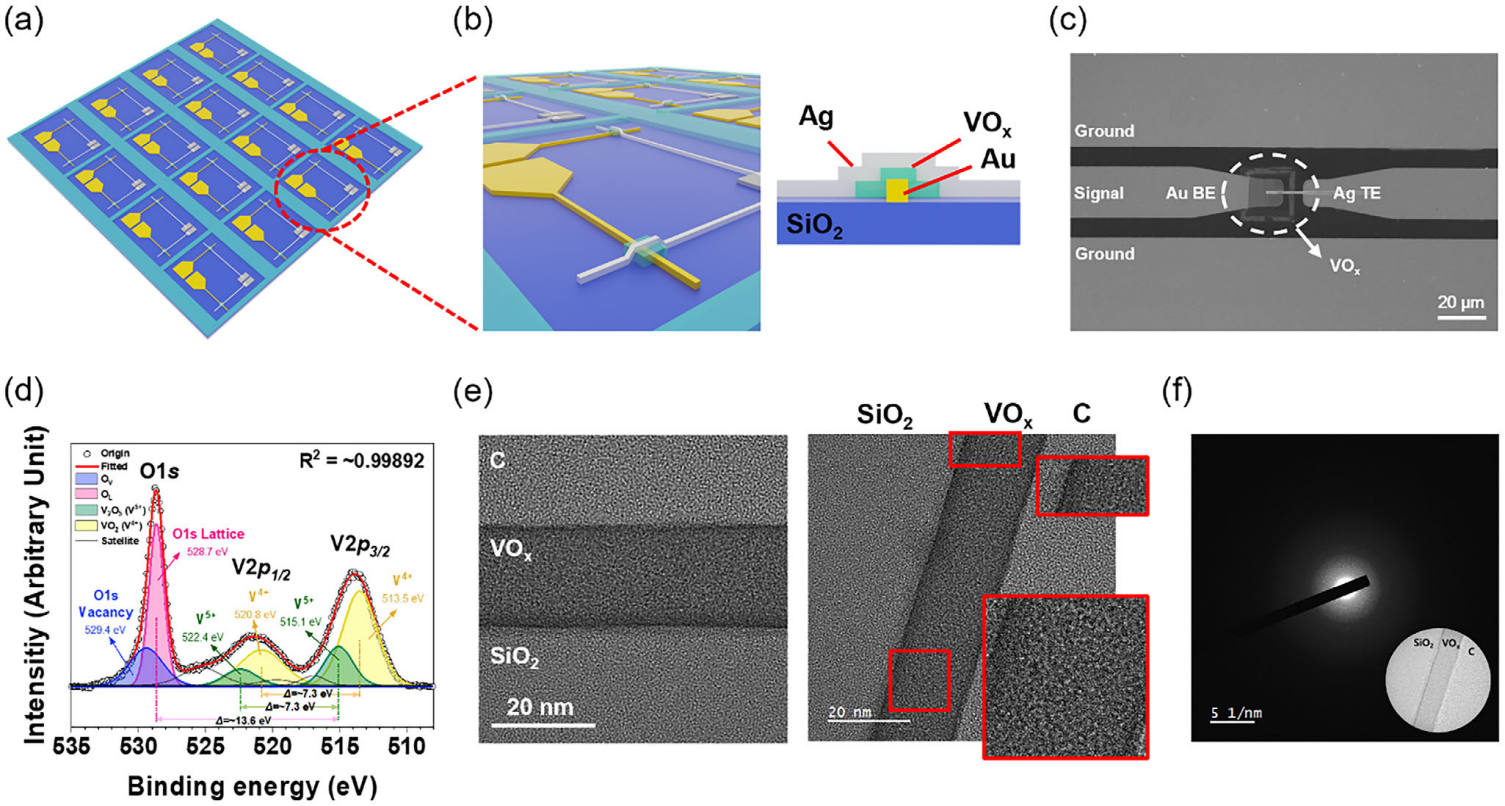
Device schematics and images with material characterization. a) Schematic illustration of the VO_x_‐based memristor crossbar array. b) Zoomed‐in perspective view and side view cross‐sectional illustration of the Ag/VO_x_/Au structure on a SiO_2_ substrate, demonstrate the overall device layout and the detailed layered configuration. c) The representative top view SEM image of the signal transmission area, and the dashed circle marks the area where the VO_x_ was deposited. d) XPS spectra of O 1s and V 2p region for the sputter‐deposited VO_x_ layer, which is etched 10 nm from the surface. e) Cross‐sectional HR‐TEM micrograph of C/VO_x_/SiO_2_ (left), and magnified VO_x_ layer in the red‐solid‐lined rectangles (right). f) SADP image of C/VO_x_/SiO_2_ layer.

VO_x_ was employed as the active layer in both memristor and RF devices, exhibiting unique multiple valence properties while forming a uniform thin film within the device structure. The 3D schematic views of the device in **Figure** **1**a,b highlight the crossbar array architecture and the metal‐insulator‐metal (MIM) configuration, respectively. Figure 1c presents a scanning electron microscopy (SEM) image of the fabricated RF switch, integrated with a ground‐signal‐ground (GSG) coplanar waveguide for RF signal transmission. Atomic force microscopy (AFM) analysis of the Ag/VO_x_/Au stack (Figure S1, Supporting Information) reveals a VO_x_ active layer thickness of ≈36 nm and an average surface roughness (*R*_a_) of 0.27 nm, indicating a smooth and uniform film. The fabrication process flow of the VO_x_ RF switch is detailed in Figure S2 (Supporting Information). Briefly, the process involves electron‐beam (E‐beam) evaporation of the bottom Au electrode onto the silicon oxide (SiO_2_) substrate, followed by sputtering and wet‐etching of the 20 µm × 20 µm VO_x_ active layer. Finally, a 100 nm‐thick top Ag feedline electrode was patterned and deposited onto the VO_x_ layer.

X‐ray photoelectron spectroscopy (XPS) analysis was performed to investigate the composition and chemical bonding states of the deposited VO_x_ layer. The O 1s spectrum revealed peaks for V─O bonds (O_L_, 528.7 eV) and oxygen vacancies (O_V_, 529.4 eV). V 2p_1/2_ and V 2p_3/2_ spectra were deconvoluted into V^5+^ and V^4+^ peaks, obtained at 522.4 and 520.8 eV for V 2p_1/2_, and 515.1 and 513.5 eV for V 2p_3/2_, respectively, with higher V^4+^ peak intensity. This indicates mixed phases of VO_2_ and V_2_O_5_, differing from stoichiometric V_2_O_5_ with dominant V^5+^ peaks.^[^ ^32^, ^33^, ^34^, ^35^, ^36^ ^]^ According to the previous report by Ryu et al.,^[^ ^29^ ^]^ rutherford backscattering spectroscopy analysis and auger electron spectroscopy of VO_x_ thin films which deposited under identical conditions in this confirmed an oxygen‐to‐vanadium ratio below 2.5, consistent with oxygen‐deficient VO_x_ layers. These oxygen vacancies facilitate Ag^+^ ion migration by lowering the activation energy and creating diffusion pathways confirmed by nudged elastic band (NEB) simulation and density functional theory (DFT) calculations, enabling fast resistive switching at low voltage.^[^ ^37^, ^38^ ^]^ More detailed XPS results, and their corresponding analysis are discussed in Note S1 (Supporting Information).

In addition to the chemical bonding states, the microstructure of VO_x_ layer was examined using transmission electron microscopy (TEM) and selected area diffraction patterns (SADPs) as shown in Figure 1e,f. For these analyses, the VO_x_ layer was deposited on a SiO_2_ substrate under the same conditions as those used for the Ag/VO_x_/Au device, except for the deposition time. An amorphous carbon (C) layer was coated on the VO_x_ thin film prior to TEM sample preparation to prevent ion beam damage during focused ion beam milling. Figure 1e shows a cross‐sectional high‐resolution transmission electron microscopy (HR‐TEM) micrograph of C/VO_x_/SiO_2_ specimen_,_ and Figure 1f shows its SADP. The HR‐TEM images confirm that the VO_x_ layer exhibits a homogeneous amorphous microstructure with an almost negligible amount of partially crystalline phases (depicted as red rectangles in Figure 1f), which is consistent with the SADPs result. In the amorphous structure of the VO_x_ layer, there are no preferential ion migration paths, such as grain boundaries in the polycrystalline layer. Therefore, it leads to reliable device operation as will be further discussed. The amorphous nature of VO_x_ layer makes it have a flat surface, consistent with the AFM result in Figure S1 (Supporting Information).^[^ ^39^, ^40^, ^41^ ^]^ This flat surface is advantageous to achieve stable device operation, particularly in filamentary‐type devices, as it minimizes the variation caused by the non‐uniform electric field in the device with rough metal‐dielectric interfaces.^[^ ^42^, ^43^ ^]^ The inherent flatness of VO_x_ mitigates these issues, ensuring reduced variability and enhanced reliability in electrical properties, as detailed in the next section.

### 2.2 DC Switching Characteristics of Ag/VO_x_/Au Device

**Figure 2:**
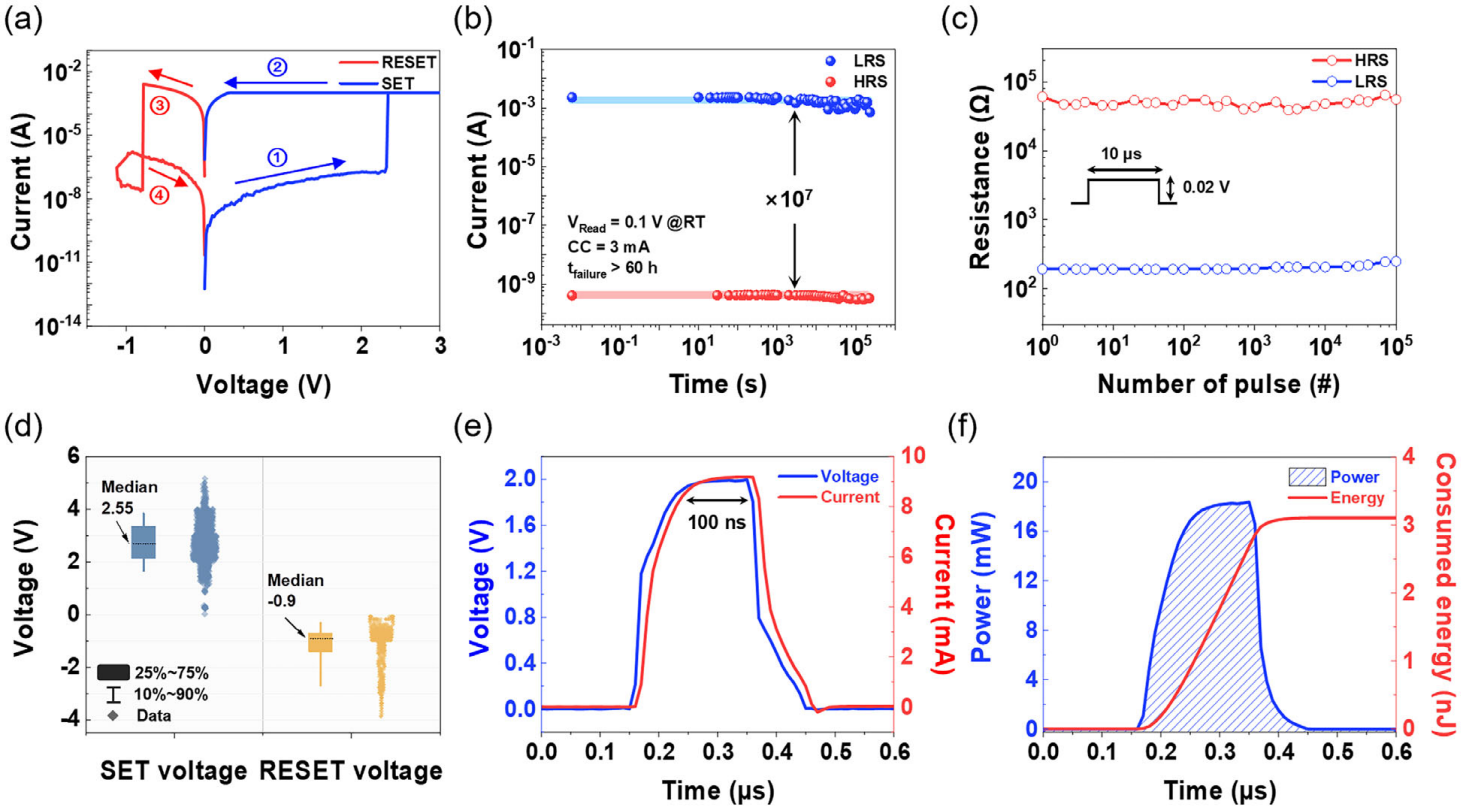
DC switching characteristics of VO_x_‐based memristor. a) Representative *I–V* curve of bipolar non‐volatile resistive switching effect in Ag/VO_x_/Au device with 4 µm × 4 µm overlap area and SET compliance current of 1 mA. Step 1: The voltage is swept from 0 to +2.4 V causes a sharp current increase to the compliance value, indicating the SET transition from HRS to LRS. Step 2: Decreasing the voltage from +3 to 0 V maintains the LRS, demonstrating non‐volatile characteristics. Step 3: The voltage decreases from 0 to −1 V. The sharp drop in current ≈−0.8 V shows the RESET process from LRS to HRS. Step 4: The voltage returns to 0 V. b) Retention performance of HRS and LRS states measured at room temperature (RT) with a +0.1 V of *V*_Read_. The device demonstrates an ON/OFF ratio of 10^7^ with a compliance current of 3 mA. Retention time exceeds 60 h, indicating stable non‐volatile characteristics. c) Read disturbance characteristics of HRS and LRS under 10^5^ read pulse stress, indicating minimal degradation and demonstrating the reliability of the resistive switching behavior. d) Distribution of the *V*_SET_ and *V*_RESET_ observed in the VO_x_ memristor switch for ≈10^3^ cycles. The median *V*_SET_ and *V*_RESET_ are +2.55 and −0.9 V, respectively. e) Nanosecond input voltage and output current pulse waveform characteristics of the VO_x_ memristor during the SET processes. The V_SET_ pulse amplitudes were set to ≈2.0 V with a pulse width of 100 ns. f) Consumed energy and power during the 100 ns SET pulse. The consumed energy is determined by integrating the power over time, yielding a measured SET energy of 3.1 nJ.

Direct current (DC) electrical characterization was performed on as‐fabricated Ag/VO_x_/Au crossbar devices, revealing their non‐volatile resistive switching behavior. The devices were SET from the HRS to the LRS by applying a voltage sweep, and the non‐volatile nature of the switching allowed the devices to retain their LRS state without continuous input power. **Figure** **2**a presents the current–voltage (*I*─*V*) characteristics of a 4 µm × 4 µm device, obtained by sweeping the voltage applied to the Ag top electrode from 0 to +3.0 V, back to 0 V, then to −1.1 V, and finally returning to 0 V, while grounding the Au bottom electrode. A compliance current of 1 and 3 mA was enforced during the SET and RESET processes to prevent permanent device breakdown. The device exhibits a stable ON/OFF ratio of ≈10^7^, as demonstrated by the retention measurements in Figure 2b. Both the LRS and HRS were maintained for over 60 h at room temperature under a read voltage of +0.1 V, indicating outstanding stability and reliability, surpassing previously reported VO_x_‐based memristor devices.^[^ ^28^, ^44^, ^45^ ^]^ Figure 2c further confirms the robustness of the device through read disturbance testing, where applying over 10^5^ read pulses of +0.02 V with a 10 µs width resulted in negligible degradation of both resistance states. Figure S3 (Supporting Information) demonstrates the high endurance of the device, maintaining a stable ON/OFF ratio over 10^3^ switching cycles. The distribution of SET voltage (*V*_SET_) and RESET voltage (*V*_RESET_) over these cycles is presented in Figure 2d, revealing median values of +2.55 and −0.9 V, respectively. These relatively higher switching voltages, compared to some previously reported VO_x_ memristor, are attributed to the crystalline structure and the overlap area of our devices.^[^ ^46^, ^47^ ^]^ As the amorphous phase of VO_x_ inherently contains a significant number of defects, and larger cells contain proportionally more defects than smaller ones, the activation energy for defect‐driven filament formation is reduced in larger cells.^[^ ^48^, ^49^ ^]^ This leads to lower switching voltages in larger devices, a trend we confirmed by fabricating a device with a 100 µm diameter using a shadow mask (Figure S4, Supporting Information). This larger device exhibited significantly reduced *V*_SET_ and *V*_RESET_ of +0.49 and −0.13 V, respectively, albeit with a reduced ON/OFF ratio of ≈100 times compared to the smaller crossbar device. This observation suggests the correlation between the overlap area, switching voltage, and ON/OFF ratio in the Ag/VO_x_/Au memristor. Figure 2e presents the rapid SET switching capability, with the device transitioning from the HRS to the LRS within a 100 ns pulse. Figure 2f reveals the corresponding power consumption and switching energy during this SET event, highlighting the energy efficiency of the switching process. A comprehensive comparison of the VO_x_ device performance against previously reported devices is provided in Table S1 (Supporting Information). Notably, our device exhibits improved or comparable performance across key metrics, including a higher ON/OFF ratio, longer retention, and greater cycling endurance. Furthermore, Figure S5 (Supporting Information) demonstrates the tunability of the ON state resistance through compliance current control. Increasing the compliance current during the SET process results in a lower ON state resistance, indicating the ability of the device to withstand high currents while achieving a desirable low‐resistance state for RF switch applications.

### 2.3 Switching and Conduction Mechanism of Ag/VO_x_/Au Memristor Devices

**Figure 3:**
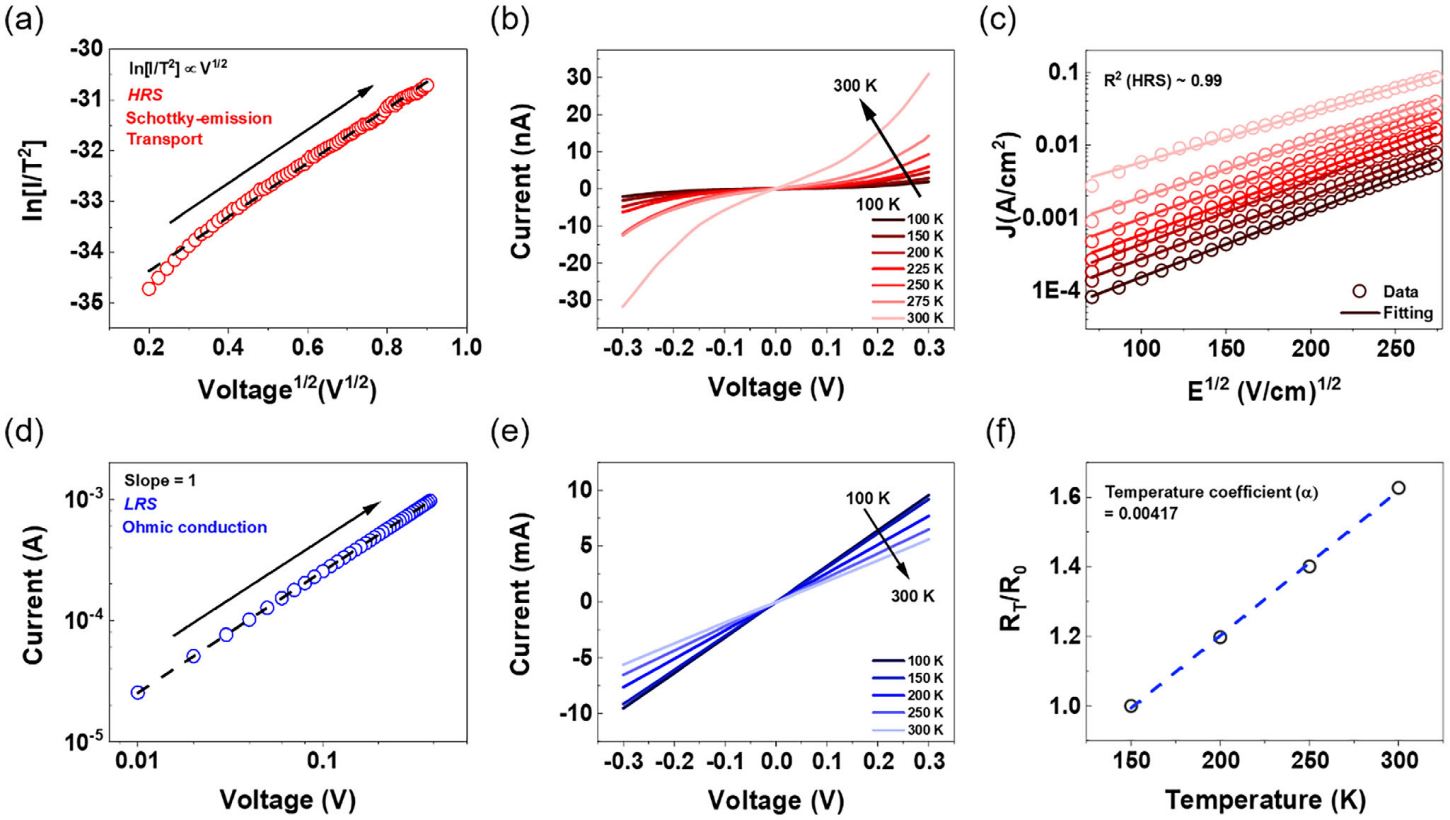
Switching and conduction mechanism of Ag/VO_x_/Au device. a) ln (*I/T* ^2^) versus *V* ^1/2^ curves in the HRS, indicating Schottky emission conduction. b,e) Typical *I*–*V* characteristics of VO_x_ memristor measured at different temperatures, with (b) showing data for the HRS with a 6 µm × 6 µm overlap area and (e) showing data for the LRS with a 2 µm × 2 µm overlap area. c) Fitted data for HRS using the Schottky emission model, demonstrating the linear relationship with the value of *R* ^2^ ≈0.99 in the logarithmic *J* versus *E* ^1/2^ plot. d) log (*I*) versus log (*V*) curves in the LRS, indicating Ohmic conduction. f) Normalized temperature‐dependent resistance in the LRS with the slope representing the *α*.

To elucidate the transport mechanism in the Ag/VO_x_/Au device, current fitting analyses and low‐temperature measurements were conducted, providing strong evidence for a filamentary conduction model involving the rapid migration of Ag^+^ ions within the VO_x_ layer. In the HRS, as shown in **Figure** **3**a, a linear relationship between ln(*I*/*T* ^2^) and *V* ^1/2^ is observed, indicative of Schottky emission. Further analysis of the *I*–*V* characteristics at varying temperatures (Figure 3b,c) reveals non‐linear *I–V* curves in the HRS, with current increasing with temperature increases, which is a typical characteristic of insulators and semiconductors. The HRS data were best fitted by the Schottky emission model, demonstrating good agreement when plotting logarithmic *J* (A/cm^2^) versus *E* ^1/2^ (V/cm)^1/2^ across all temperatures. The Schottky barrier height was calculated using Equation (1):^[^ ^50^ ^]^

(1) $$\begin{array}{c} J=A^{*}\;T^{2}exp\left[\frac{-q\left(\phi_{B}-\sqrt{\frac{qE}{4\pi \varepsilon_{r}\varepsilon_{0}}}\right)}{kT}\right]\\ \;A^{*}=\frac{120m^{*}}{m_{0}}\;\; \end{array}$$

where *J* is the current density, A^*^ is the effective Richardson constant, *m_0_* is the free electron mass, m^*^ is the effective mass, *q* is the electron charge, *T* is the absolute temperature, *E* is the electric field across the dielectric, *k* is Boltzmann's constant, *ε*_0_ is the permittivity of free space, and *ε*_r_ is the relative dielectric constant. Using an effective thickness of ≈40 nm and assuming *m* ^*^/*m*_0_ ≈ 1, the extracted barrier height (*ϕ*_B_) is ≈0.245 eV at 300 K, based on the relative dielectric constant of VO_x_ (*ε*_r_ = 14.6) measured from capacitance–voltage (*C*─*V*) (Figure S6, Supporting Information). In the LRS, the double‐logarithmic *I*–*V* plot (Figure 3d) exhibits a slope of ≈1, indicative of Ohmic conduction behavior resulting from the formation of conductive filaments. The decrease in LRS current with increasing temperature (Figure 3e) suggests metallic‐like conduction within the filament. To further characterize the nature of these filaments, the temperature coefficient of resistance in the LRS was determined using Equation (2):^[^ ^51^ ^]^

(2) $$R\;\left(T\right)=R_{0}\left[1+\alpha \left(T-T_{0}\right)\right]\;$$

where *R*_0_ is the resistance at a reference temperature *T*_0_, and *α* is the temperature coefficient of resistance. Linear fitting of the normalized LRS resistance (*R*_T_/*R*_0_) as a function of temperature (Figure 3f) yields a temperature coefficient of resistance (*α*) of 4.2 × 10^−3^ K^−1^ for the Ag/VO_x_/Au device. This value closely matches the reported *α* of Ag (4.1 × 10^−3^ K^−1^), strongly suggesting that the conductive filaments are primarily composed of Ag, formed by the diffusion of Ag^+^ ions from the top electrode into the VO_x_ layer.^[^ ^52^ ^]^

Building on these findings, the conduction mechanism can be further understood by examining the interface properties. Considering that the work function of n‐type VO_x_ typically ranges from 4.6 to 5.02 eV, the higher work function of the Au electrode (5.1 eV) is consistent with the formation of a Schottky barrier at the VO_x_/Au interface.^[^ ^53^, ^54^, ^55^ ^]^ Conversely, the lower work function of the Ag electrode (4.32 eV) suggests the formation of an Ohmic contact at the Ag/VO_x_ interface, aligning with the observed experimental *I*–*V* characteristics.^[^ ^56^ ^]^

### 2.4 Radio‐Frequency Characterization of VO_x_ RF Switches

The high‐frequency performance of the VO_x_ RF switch was evaluated by measuring its scattering parameters (S‐parameters) in both the ON and OFF states using a vector network analyzer (VNA). Accurate measurements were ensured by performing an on‐wafer short‐open‐load‐through (SOLT) calibration to eliminate the influence of the test cables and probe station. The calibration accuracy was verified by measuring the calibrated S_11_ and S_21_ of reflection standards, as shown in Figures S7–S10 (Supporting Information). To extract the intrinsic S‐parameters of the VO_x_ RF switch, de‐embedding procedures were performed using on‐chip test patterns to remove the contributions of probe‐pad and interconnect parasitics.

**Figure 4:**
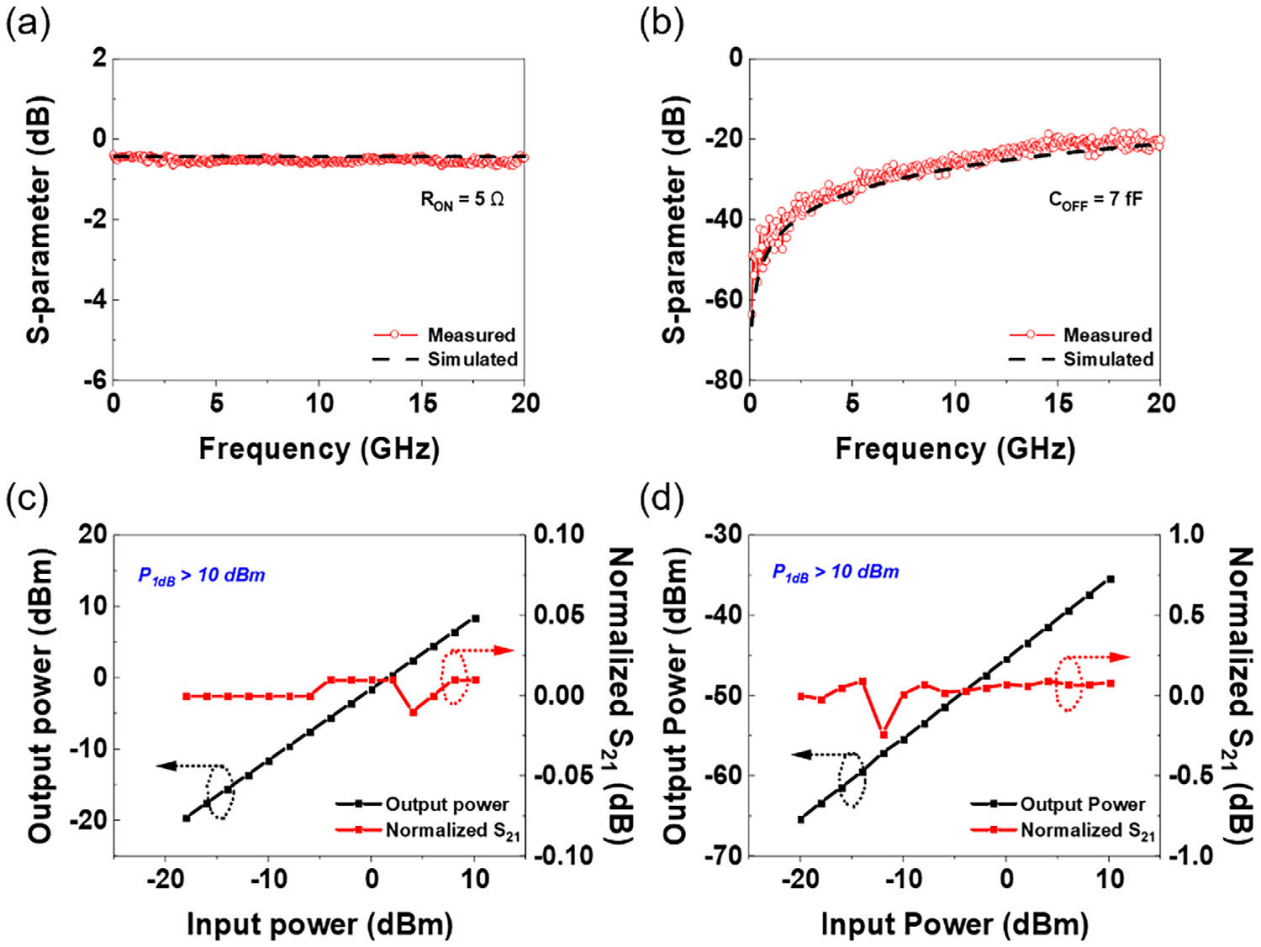
Radio‐frequency characterization of VO_x_ RF switches. Experimental S‐parameter data in both the a) ON state and b) OFF state Ag/VO_x_/Au structured RF switches with an overlap area of 4.3 µm × 0.5 µm with measurements up to 20 GHz. The VO_x_ RF switch was measured with a current compliance of 10 mA. The values of *C*_OFF_ (7 *f*F) and *R*_ON_ (5 Ω) were extracted using a lumped element equivalent circuit model. Calculated from this, the cutoff frequency is 4.5 THz. The insertion loss at 20 GHz in the ON state was 0.455 dB, and the isolation at 20 GHz in the OFF state was 20.032 dB. The dashed lines are the simulated data from the equivalent circuit model. Power‐handling data of VO_x_‐based RF switches, with c) representing the ON state and d) the OFF state. The black line shows the output power on a dBm scale, while the red line shows the normalized S_21_ on a dBm scale as a function of input power. In both states, no 1 dB power compression occurred at input power above 10 dBm, the measurement limit of the VNA source.

**Figure** **4**a,b present the de‐embedded S_21_ results for Ag/VO_x_/Au devices with an overlap area of 4.3 µm × 0.5 µm and switching with a compliance current of 10 mA. The VO_x_ switch demonstrates excellent RF performance, exhibiting an insertion loss of only 0.45 dB in the ON state and an isolation of 20 dB in the OFF state up to 20 GHz. An equivalent lumped element circuit model (Figure S11, Supporting Information) was used to extract the key performance parameters: ON state resistance (*R*_ON_), OFF state capacitance (*C*_OFF_), and cutoff frequency (*F*_CO_). From the de‐embedded S‐parameters, *R*_ON_ and *C*_OFF_ were determined to be 5 Ω and 7 *f*F, respectively, resulting in a calculated *F*_CO_ of ≈4.5 THz based on the relation *F*_CO_ = 1/2*πR*_ON_ *C*_OFF_.^[^ ^57^ ^]^ To assess the applicability of the switch at a higher frequency, measurements were extended up to 67 GHz (Figure S12, Supporting Information), demonstrating reliable performance in this regime and emphasizing its potential for integration into advanced mmWave technologies. A benchmark comparison of various RF switch technologies (Table S2, Supporting Information) reveals that the VO_x_ switch offers comparable or superior performance to PCM and microelectromechanical system (MEMS) switches, with the added advantages of heater‐less ambient integration and low switching voltage. Notably, it exhibits a higher ON/OFF ratio and cutoff frequency among oxide‐based RF switches.

High‐power handling capability is crucial for system‐level applications of RF switches. Therefore, the 1 dB compression point (*P*_1dB_) was evaluated through power‐dependent loss measurements. Figure 4c,d show the power handling characteristics of a 2 µm × 2 µm VO_x_ RF switch at 40 GHz for input power levels ranging from −20 to +10 dBm. In the ON state, the output power exhibits a linear relationship with the input power, with no observable output power compression (> 0.01 dB) up to an input power of +10 dBm. In the OFF state, the switch maintains high isolation, with output power exhibiting losses exceeding 35 dB and remaining linear for input powers up to +10 dBm.

### 2.5 Reconfigurable Bandpass Filter Using VO_x_ RF Switch

The increasing demand for compact, low‐loss, and reconfigurable bandpass filters (BPFs) in microwave and RF communication systems has driven the exploration of novel switching technologies.^[^ ^58^, ^59^, ^60^ ^]^ Reconfigurable BPFs offer dynamic frequency tuning through external control, leading to benefits such as improved signal‐to‐noise ratio and enhanced out‐of‐band performance.^[^ ^61^ ^]^ While various approaches for frequency tuning have been proposed, including RF MEMS switches, PIN diodes, and ferroelectric capacitors, they often suffer from limitations in switching speed, power consumption, or integration complexity.^[^ ^62^, ^63^, ^64^ ^]^

**Figure 5:**
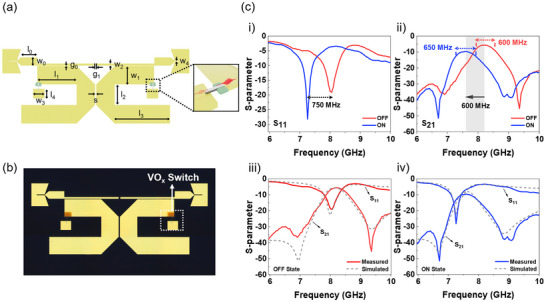
The typical measured and simulated S‐parameters of the filter are presented for both switch states. a) Schematic diagram of the X‐band filter with key dimensions labeled and b) optical image of the fabricated filter. The dashed box indicates the VO_x_ memristor switches with an overlap area of 5 µm × 30 µm. c) The typical measured and simulated S‐parameters of the filter in both switch states: i) Measured S_11_ and ii) S_21_ showing the tunable bandpass filter using VO_x_ switches in ON and OFF states. iii) Comparison of measured and simulated S‐parameter in the OFF state, and iv) in the ON state, with the dashed lines representing simulation results obtained from ADS.

This work presents a novel reconfigurable X‐band BPF employing VO_x_ memristor devices integrated within a 50‐µm‐pitch GSG configuration. This design leverages the fast switching speed and non‐volatility of the VO_x_ switch to achieve zero‐static power consumption, offering a distinct advantage over conventional approaches.^[^ ^65^ ^]^ The filter, optimized using the Advanced Design System (ADS) simulator, was fabricated on a 430 µm‐thick sapphire substrate (*ε_r_* = 11.6). **Figure** **5**a,b show the schematic and optical image of the compact 10.8 mm × 0.9 mm filter, respectively. The total resonator length (*l*_1_ + *l*_2_ + *l*_3_) is 5.62 mm, the coupling length (*l*_2_) is 1 mm, and the gap between the feed lines and the resonators (*g*_0_) is 0.015 mm. Detailed information on the fabrication process and structural dimensions is provided in Figure S13 (Supporting Information). The integrated VO_x_ switches exhibit stable and low‐power operation, as demonstrated by the DC *I–V* curves in Figure S14 (Supporting Information), showing consistent characteristics over 100 cycles with both *V*_SET_ and *V*_RESET_ within ±1 V.

Figure 5c presents the measured and simulated S‐parameters of the filter in both the ON and OFF states, demonstrating its reconfigurable behavior. In the OFF state, the filter operates at a center frequency of 8.2 GHz (simulated: 8.15 GHz) with an insertion loss of 5.6 dB, a 3‐dB bandwidth of 600 MHz, and a return loss of 20.2 dB. In the ON state, the center frequency shifts to a lower value of 7.6 GHz (simulated: 7.6 GHz), with an insertion loss of 9.6 dB, a bandwidth of 650 MHz, and a return loss of 28.2 dB. The quality factor (*Q*) of resonators with VO_x_ switches, estimated using Equation (3), is 112.3 in the OFF state and 89.4 in the ON state.^[^ ^66^ ^]^

(3) $$Q=\frac{f_{c}}{FBW}\;$$

*f*_c_ is the center frequency and *FBW* is fractional bandwidth.

The measured center frequency closely match the ADS simulation results, validating the filter design. Further optimization of parameters such as substrate permittivity and metal conductivity can enhance accuracy and reliability (Note S2, Supporting Information). However, considering the complex interactions within the filter structure, more in‐depth theoretical studies are warranted to fully elucidate the underlying physics. Figure S15 (Supporting Information) illustrates the simulated surface current density distribution, highlighting the activation of the parasitic patch through the connection between the main patch and the parasitic patch, demonstrating the principle of frequency tuning via switch integration. Table S3 (Supporting Information) benchmarks the performance of this VO_x_‐based BPF against other reported X‐band filters, emphasizing its superior advantages of zero‐static power dissipation due to the non‐volatile nature of the VO_x_ switches, coupled with fast switching speed.

## 3 Conclusion

**Figure 6:**
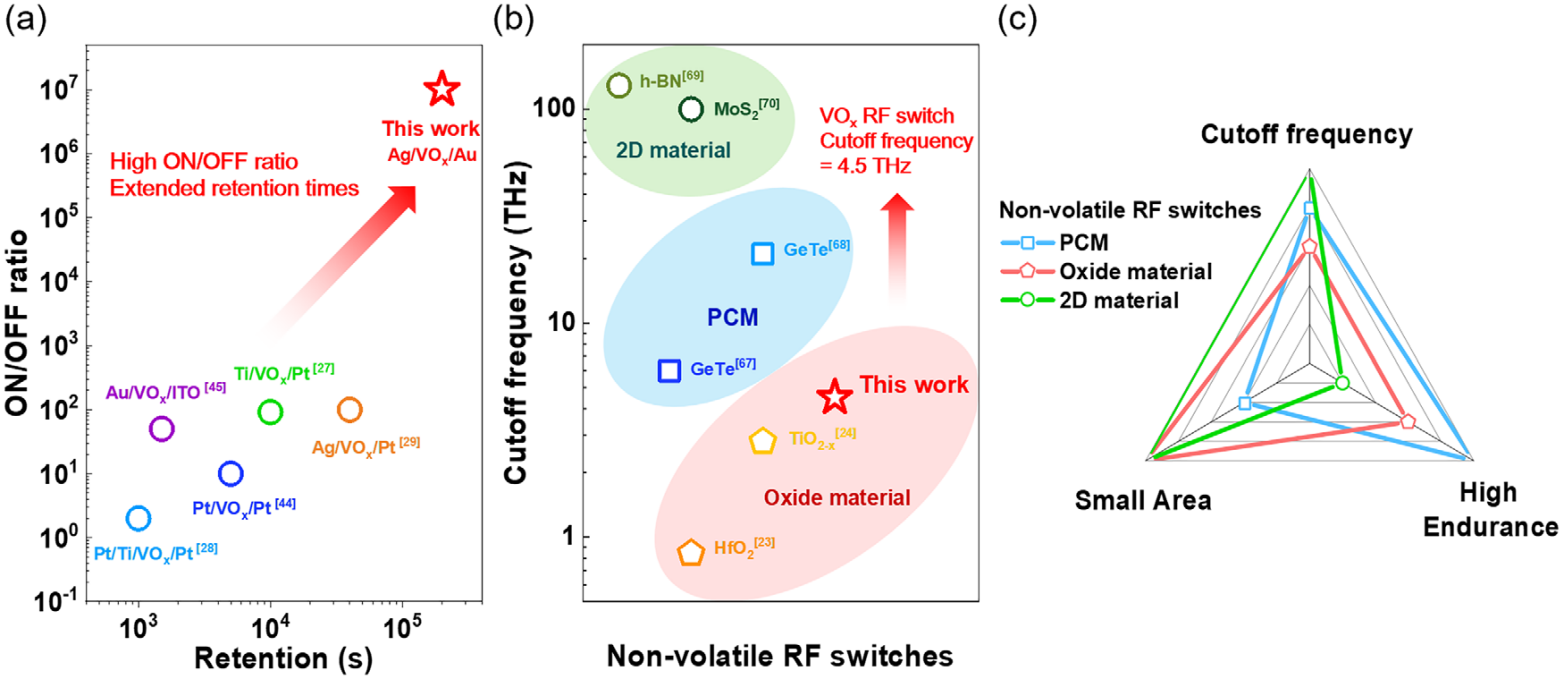
a) Benchmarking the ON/OFF ratio and retention times of VO_x_ memristor against other reported VO_x_‐based devices. The plotted data highlight the outstanding performance of this work, achieving a significantly higher ON/OFF ratio (≈10^7^) and extended retention times (>10^5^ s) compared to previously reported devices with different electrode configurations.^[^ ^27^, ^28^, ^29^, ^44^, ^45^ ^]^ b) Cutoff frequency comparison of VO_x_ RF switches with various non‐volatile RF switch technologies. The green region represents 2D‐based RF switches, the blue region corresponds to PCM‐based RF switches, and the red region denotes oxide‐based RF switches. The highlighted performance of this work demonstrates a VO_x_ RF switch achieving a cutoff frequency of 4.5 THz, showing superior potential in oxide‐based RF switches.^[^ ^23^, ^24^, ^67^, ^68^, ^69^, ^70^ ^]^ c) Radar chart of representative non‐volatile RF switching materials. While exhibiting a superior cutoff frequency, 2D materials are limited by low cycling endurance; similarly, PCM's scalability is constrained by its heater requirement. Oxide materials, in contrast, demonstrate a well‐balanced performance across all key metrics.

In conclusion, this work has successfully demonstrated the development of nanoscale, non‐volatile RF switches based on VO_x_ for next‐generation wireless communication systems. The fabricated Ag/VO_x_/Au switches exhibit robust non‐volatile bipolar resistive switching behavior, with a high ON/OFF ratio of ≈10^7^ and switching voltages below 3 V. Furthermore, the VO_x_ switches demonstrate rapid switching with pulse operation, consuming a maximum power of less than 18.5 mW and an energy below 3.2 nJ. Advanced frequency characterization techniques, including precise calibration and de‐embedding, reveal outstanding RF performance, including an exceptionally low insertion loss of 0.45 dB and isolation exceeding 20 dB across a wide frequency range. The practical application of these switches was demonstrated through their integration into a reconfigurable X‐band filter, achieving tunable frequency with fast switching speeds and, crucially, zero‐static power dissipation due to the non‐volatile nature of the VO_x_ memristor. As shown in **Figure** **6**, our device demonstrates a 4 times improvement in retention and a 10^5^ times increase in ON/OFF ratio compared to previously reported VO_x_‐based memory, achieved through contact metal engineering and device structure optimization. Furthermore, the device exhibits a 60% higher cutoff frequency compared to previously reported oxide‐based RF switches. To further enhance device performance, additional studies on the optimization and analysis of VO_x_ film characteristics are required. These findings underscore the significant potential of our device as high‐performance, energy‐efficient, nanoscale switches for future reconfigurable RF front‐end systems in advanced wireless communication applications.

## 4 Experimental Section

### Device Fabrication and Characterization

The VO_x_ memristor and RF switches were fabricated on a SiO_2_ substrate, with the memristor patterned in a crossbar array structure and the RF switches patterned in a 50‐µm‐pitch GSG configuration, both using photolithography. An E‐beam evaporator was used to deposit the ground pads and the bottom electrode consisting of 2‐nm‐thick Cr for the adhesion layer and 60‐nm‐thick Au. Then, a 40‐nm‐thick VO_x_ layer was deposited onto the entire wafer by RF magnetron sputtering using a high‐purity VO_2_ target (V:O = 1:2) with a diameter of 2 inches. The deposition was conducted at room temperature, with a base pressure in the low 10^−6^ Torr range and a working pressure of 20 mTorr under an Ar atmosphere (20 sccm). The RF power was maintained at 150 W during the deposition. The VO_x_ active layer was patterned by photolithography, followed by an etching process. To ensure a small area of the top electrode, it was patterned by electron beam lithography (EBL) and deposited by E‐beam evaporator of 2‐nm‐thick Cr for the adhesion layer and 100‐nm‐thick Ag. The structure of the device was analyzed via high‐resolution transmission electron microscopy (HR‐TEM; JEM‐2100F, JEOL). The compositions and chemical bonding states of the VO_x_ layer were examined through X‐ray photoelectron spectroscopy (XPS; K‐alpha, ThermoFisher). XPS was performed using a monochromated Al‐Kα source (*hν* = 1486.6 eV), with the system featuring a micro‐focused monochromator and a double‐focusing hemispherical analyzer with a 128‐channel detector. The experimental XPS spectra were deconvoluted using the CasaXPS software. Scanning electron microscopy images were collected on a (Cold FE‐SEM; S‐4800, Hitachi High‐Technologies) instrument with the beam energy at 5 kV.

### Bandpass Filter Fabrication

The 1.0 cm × 1.0 cm area of a 430 µm‐thick sapphire substrate was dried by a nitrogen gun after rinsing with acetone, and isopropyl alcohol (IPA). The 360 nm‐thick Au electrode was deposited on the sapphire substrate and patterned by a photolithography system. To minimize the undercutting of the etchant into the metal, inductively coupled plasma‐reactive ion etching (ICP‐RIE) was performed for 120 s using Cl_2_ gas, after which etching was performed using Au etchant and Cr etchant. The 300 µm × 400 µm area VO_x_ active layer was deposited by the RF magnetron sputtering with a shadow mask. The top electrode, consisting of a 2‐nm‐thick Cr adhesion layer and a 100‐nm‐thick Ag layer, was patterned by EBL to form a 5 µm × 30 µm overlap area with the VO_x_/Au stack, followed by deposition using an E‐beam evaporator.

### DC Measurements

The electrical characteristics of the device including resistive switching, endurance properties, voltage pulse measurements, and read pulse disturbance measurements were examined using a Keysight B1500A semiconductor parameter analyzer with a B1530 waveform generator/fast measurement unit pulsing module under ambient conditions. For the voltage pulse measurements, a rectangular pulse with a rising time of 100 ns, a pulse width of 100 ns at an amplitude of 2 V, and a falling time of 100 ns was applied to the device. The low‐temperature measurements were conducted using a Keithley 4200‐SCS semiconductor parameter analyzer from 100 to 300 K under vacuum conditions with the chamber pressure maintained at 10^−6^ torr. In the measurement process, the voltage was applied to the top Ag electrode while the bottom Au electrode was grounded.

### RF Measurements

The Formfactor Infinity GSG probes and the Keysight P5024A‐402 VNA was used for on‐wafer measurements from 10 MHz to 20 GHz, while the Keysight N5247A VNA was employed for measurements extending up to 67 GHz to evaluate higher frequency operation. The RF power was set to a nominal level of −20 dBm for small‐signal measurements. Before the actual device measurement, a Formfactor 138–356 Impedance Standard Substrate and WinCal software (FormFactor Inc.) were used to perform the SOLT calibration. Due to the non‐volatile resistive switching of the VO_x_ switch device, a forward and reverse DC bias was employed to turn the switching devices ON and OFF. S‐parameters were measured for each device in both the ON and OFF states, and parameters were extracted from the de‐embedded S‐parameters to determine the ON and OFF state equivalent lumped circuit component models. A de‐embedding process utilizing an open‐through configuration was employed to eliminate the pad and interconnect resistances. The power handling capability was evaluated in both the ON and OFF states by measuring the S‐parameters as the devices were incrementally subjected to RF power levels ranging from −20 to +10 dBm.

## Conflict of Interest

The authors declare no conflict of interest.

## Author Contributions

D.S. and D.K. contributed equally to this work. D.S., D.K. and J.R. performed all the material characterizations, device fabrications, DC/RF measurements, and electromagnetic simulations. C.P. and S.L. performed data curation and formal analysis. D.S. and M.K. examined all of the electrical data and prepared the manuscript, with help from the other collaborators. M.K. coordinated and supervised the research.

## Supporting information



Supporting Information

## Data Availability

The data that support the findings of this study are available from the corresponding author upon reasonable request.
