# Supplementary material for: VOx‐Based Non‐Volatile Radio‐Frequency Switches for Reconfigurable Filter
Source: Adv Sci (Weinh). 2025 May 28;12(31):e01989. doi: 10.1002/advs.202501989 (PMC12376588; doi:10.1002/advs.202501989)
Supplement: Supplementary file 1 — Supporting Information [file ADVS-12-e01989-s001.docx]

**VO_x_-based Non-Volatile Radio-Frequency Switches for Reconfigurable Filter**

*Dabin Seo^1+^, Dahyeon Kim^2+^, Jiyeon Ryu^1^, Changwoo Pyo^1^, Seungchan Lee^2^, Tae-Sik Yoon^1,3^, and Myungsoo Kim^1,2^**

^1^Graduate School of Semiconductor Materials and Devices Engineering, Ulsan National Institute of Science and Technology (UNIST), Ulsan 44919, South Korea.

^2^Department of Electrical Engineering, Ulsan National Institute of Science and Technology (UNIST), Ulsan 44919, South Korea.

^3^Department of Materials Science and Engineering, Ulsan National Institute of Science and Technology (UNIST), Ulsan 44919, South Korea.

^*^Corresponding authors Email: myungsookim@unist.ac.kr (M.K.)

**Supporting Figure 1 | AFM analysis of VO_x_ surface morphology and thickness in Ag/VO_x_/Au structures.**

**Supporting Figure 2 | Fabrication process of the Ag/VO_x_/Au RF switch structure.**

**Supporting Figure 3 | VO_x_ memristor DC endurance characteristic.**

**Supporting Figure 4 | VO_x_ memristor switching voltage characteristics for large overlap area.**

**Supporting Figure 5 | *I*–*V* characteristics of VO_x_ switches under different compliance current conditions.**

**Supporting Figure 6 | VO_x_ electrical dielectric constant (*ε*_r_) calculation through *C*–*V* measurements.**

**Supporting Figure 7 | After-calibrated S_11_ and S_21_ result of a Short standard.**

**Supporting Figure 8 | After-calibrated S_11_ and S_21_ result of an Open standard.**

**Supporting Figure 9 | After-calibrated S_11_ and S_21_ result of a Load standard.**

**Supporting Figure 10 | After-calibrated S_11_ and S_21_ result of a Through standard.**

**Supporting Figure 11 | Lumped element equivalent circuit model of RF switch.**

**Supporting Figure 12 | Performance evaluation of Ag/VO_x_/Au RF switches in the 67 GHz frequency range.**

**Supporting Figure 13 | Fabrication process flow and component dimensions of the reconfigurable X-band Filter.**

**Supporting Figure 14 | Typical DC cycling of VO_x_-based non-volatile resistive switches integrated within a filter structure.**

**Supporting Figure 15 | Simulated current density distribution of the filter model.**

**Supporting Note 1 | Detailed results and discussion of XPS analysis.**

**Supporting Note 2 | ADS simulation.**

**Supporting Table 1 | Non-volatile resistive switching performance comparison of VO_x_-based memristor.**

**Supporting Table 2 | RF switch technologies comparison table**

**Supporting Table 3 | Performance comparison of the X-band reconfigurable filter.**

**Supporting figures**

**
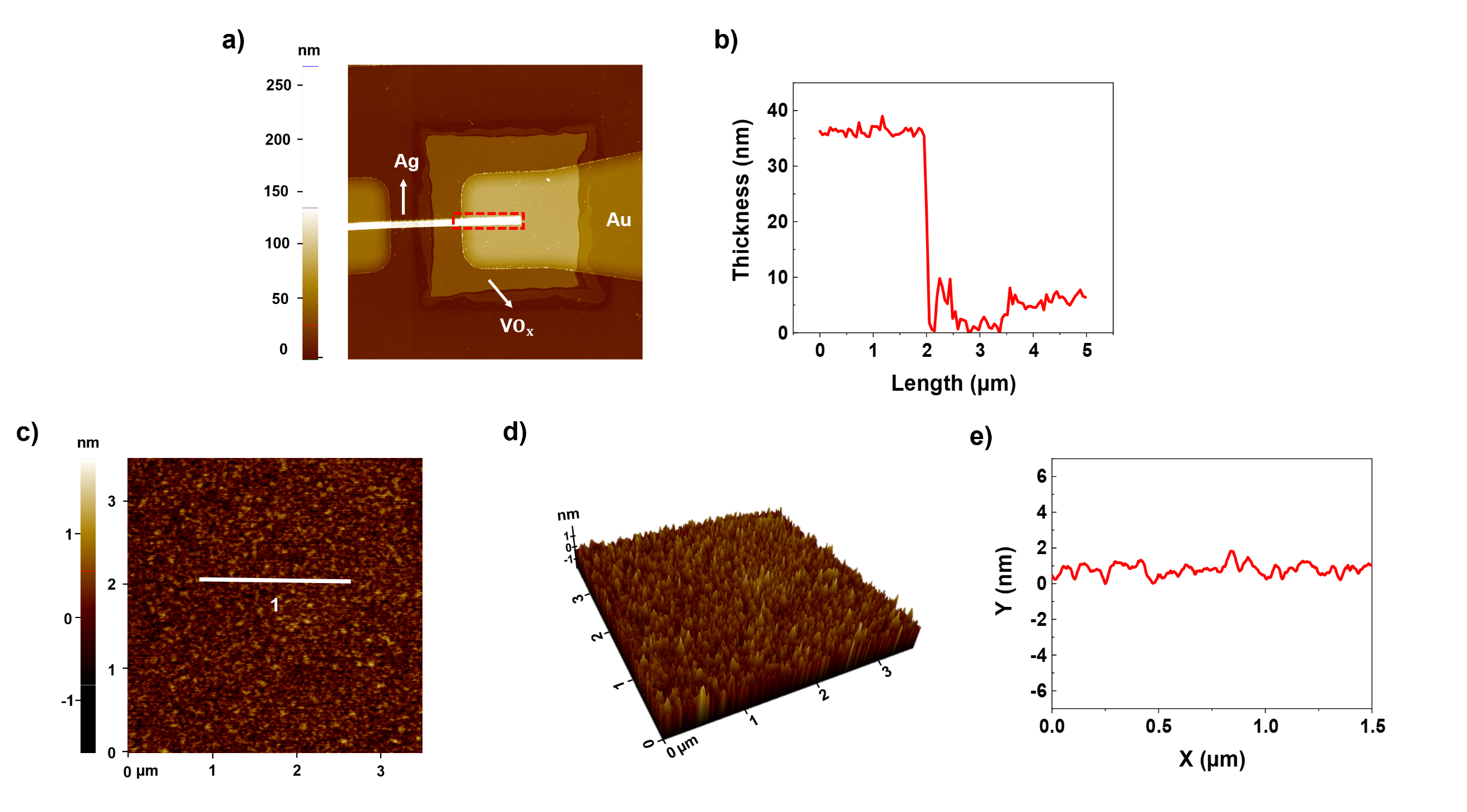
**

**Supporting Figure 1 | AFM analysis of VO_x_ surface morphology and thickness in Ag/VO_x_/Au structures.** (a) AFM images of the Ag/VO_x_/Au structure, with the dashed box indicating the overlap area. The overlap area was quantitatively extracted from the AFM measurements and determined to be 5.796 µm^2^. (b) The thickness profile of the VO_x_ switching layer shows a thickness of approximately 36 nm. (c) Surface topography and (d) 3D morphology of the VO_x_ layer. The surface has a low average roughness (*R*_a_) of 0.27 nm across a 3.5 µm × 3.5 µm area. (e) Extracted profile along line 1 from c, demonstrating the nanometer-scale uniformity of the VO_x_ surface.

**
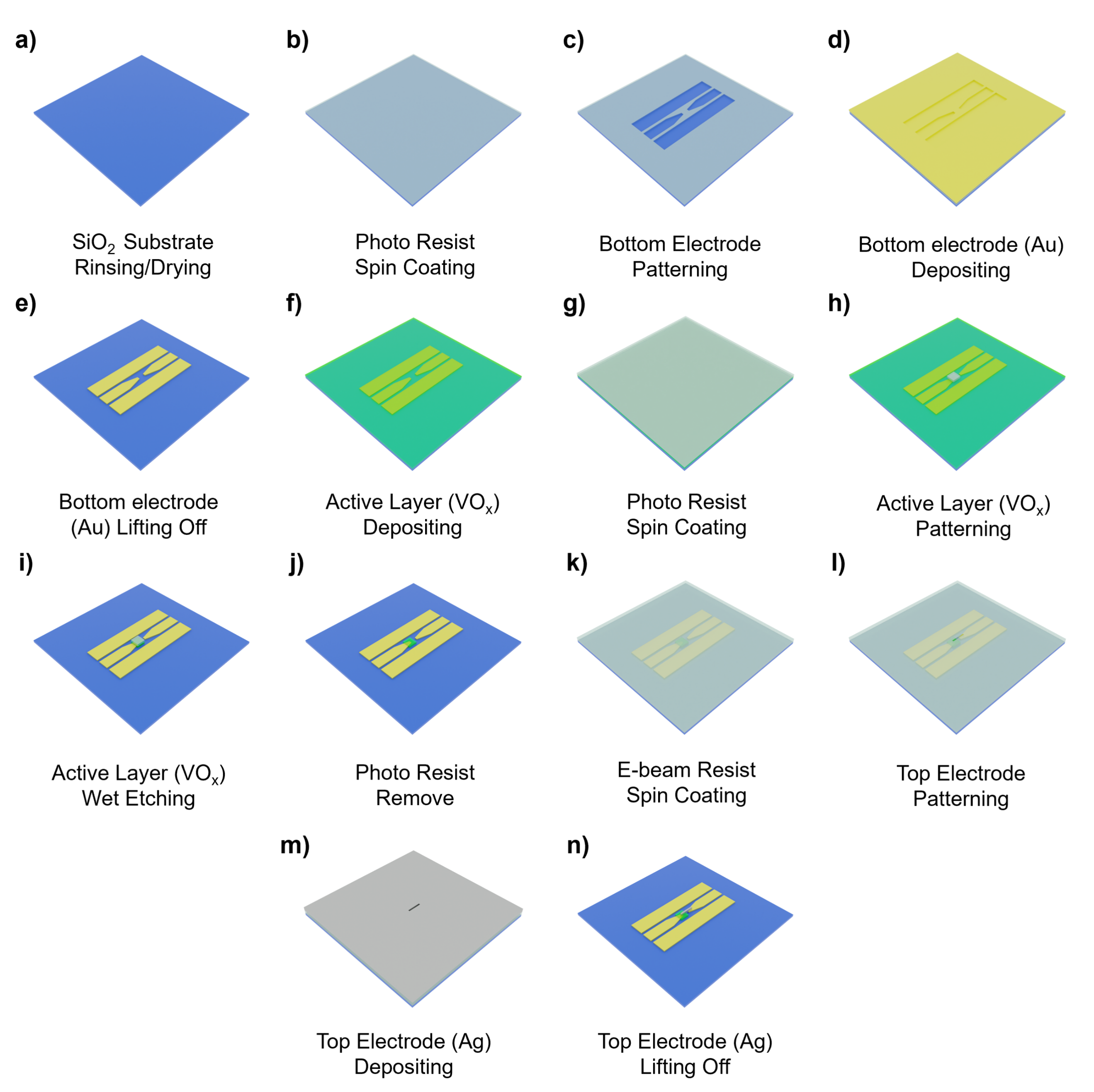
**

**Supporting Figure 2 | Fabrication process of the Ag/VO_x_/Au RF switch structure.** (a) The SiO_2_ substrate was rinsed with acetone/IPA and dried with the nitrogen spray gun. (b) The photoresist was spin-coated on the SiO_2_ substrate. (c) The bottom electrode was patterned by the photolithography system. (d) The Au metal was deposited on the lithographed bottom electrode. (e) The rest of the photoresist was lifted off from the SiO_2_ substrate. (f) The VO_x_ active layer was deposited on the SiO_2_ substrate Au electrode through RF magnetron sputtering. (g) The photoresist was spin-coated on the SiO_2_ substrate, which was covered by the active layer. (h) The photolithography system patterned the active layer. (i) The rest of the active layer was wet-etched. (j) The photoresist was removed from the SiO_2_ substrate. (k) The E-beam resist was spin-coated on the SiO_2_ substrate. (l) The top electrode was patterned using an EBL system. (m) The Ag metal was deposited on the lithographed top electrode. (n) The rest of the E-beam resist was lifted off from the SiO_2_ substrate.


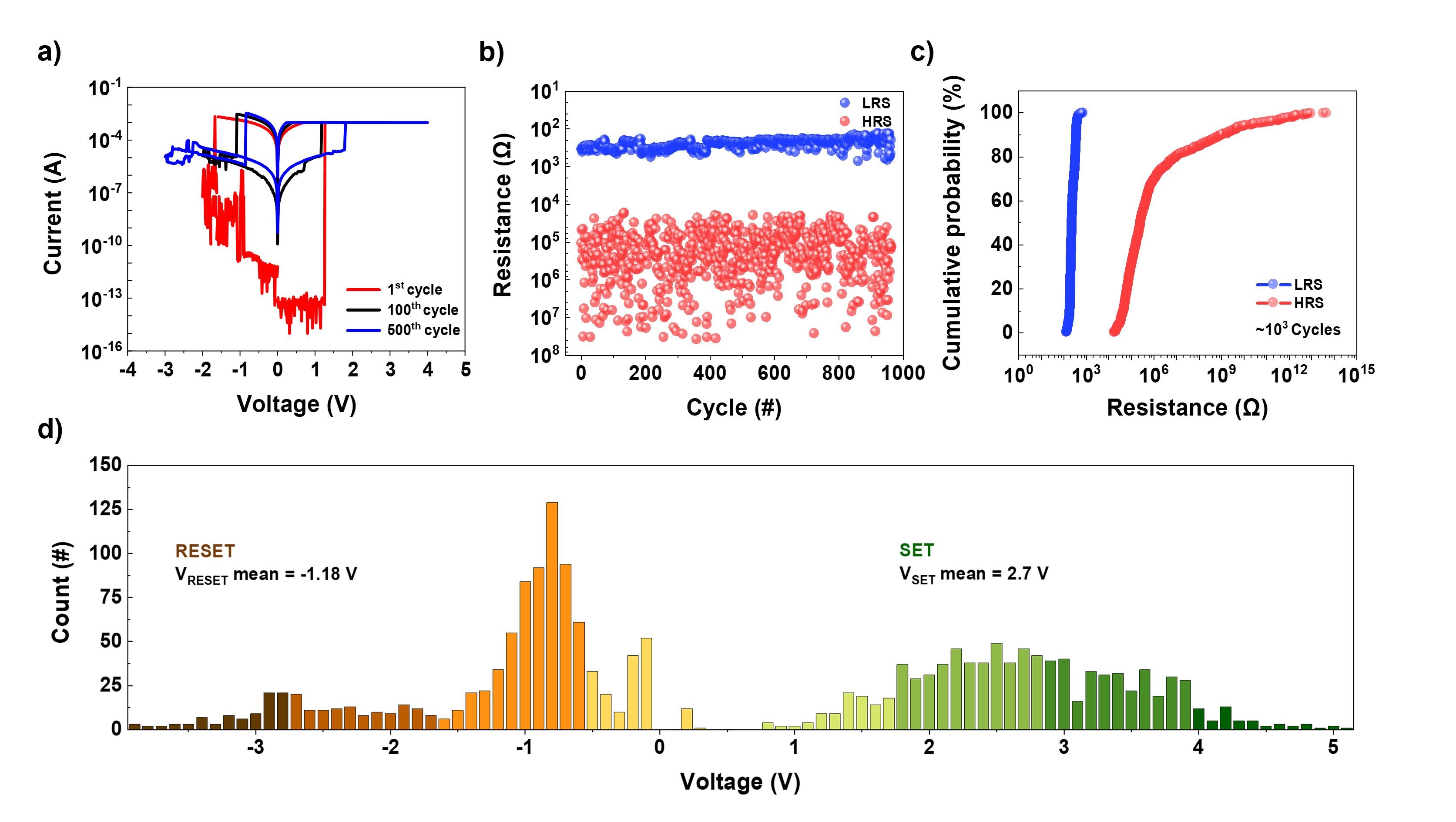


**Supporting Figure 3 | VO_x_ memristor endurance characteristic.** (a) DC *I***–***V* sweep curves of the Ag/VO_x_/Au device, corresponding to the 1^st^, 100^th^, and 500^th^ cycles. (b) Endurance characteristics of the VO_x_ crossbar MIM device with DC switching cycles. The resistance ratio consistently remained above 10 throughout all cycles, with a minimum ON/OFF ratio of 10 and a median value of 10^2^. (c) The cumulative probability function of the cycle-to-cycle distribution of the current level shows that LRS exhibits less variation than HRS. (d) Distribution of *V*_SET_ and *V*_RESET_ over approximately 1000 successive cycles. The mean values of *V*_SET_ and *V*_RESET_ are +2.7 V and −1.18 V, respectively.


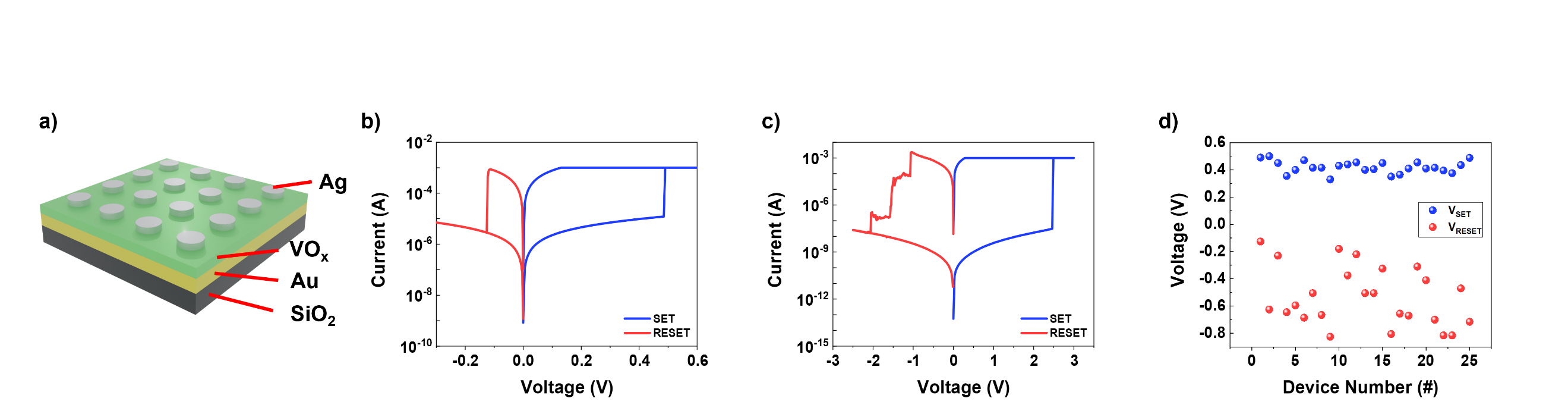


**Supporting Figure 4 | VO_x_ memristor switching voltage characteristics for large overlap area.** (a) Schematic illustration of the Ag/VO_x_/Au device. The top Ag electrode was deposited by E-beam evaporation through a shadow mask with a diameter of 100 µm. (b) Representative *I*–*V* curve of resistive switching VO_x_ device with a top electrode diameter of 100 µm. (c) Representative *I*–*V* curve of a crossbar array structure with a smaller overlap area of 2 µm × 2 µm. Compared to the device with a 100 µm top electrode, the crossbar device exhibits a higher switching voltage but achieves an ON/OFF ratio approximately 100 times greater. (d) Device-to-device distribution of *V*_SET_ and *V*_RESET_ was obtained in 25 randomly picked devices.


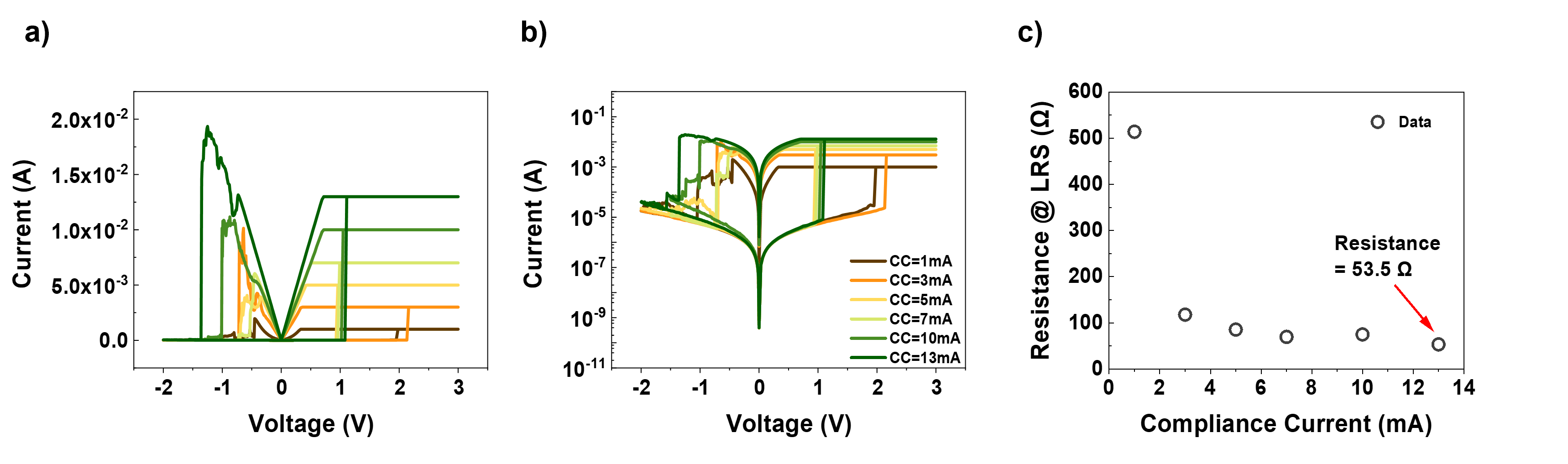


**Supporting Figure 5 | *I*–*V* characteristics of VO_x_ switches under different compliance current conditions.** (a, b) Representative *I*–*V* characteristics of the bipolar resistive switching behavior in a VO_x_-based MIM device, presented in both linear and logarithmic scales. The DC cycling measurements were performed under compliance currents of 1, 3, 5, 7, 10, and 13 mA. The VO_x_ memristor features an overlap area of 8 µm × 8 µm. (c) The ON state resistance demonstrates an exponential reduction as the applied DC compliance current increases. At a compliance current of 13 mA, the resistance reaches 53.5 Ω, demonstrating its potential suitability for RF switch applications.

_
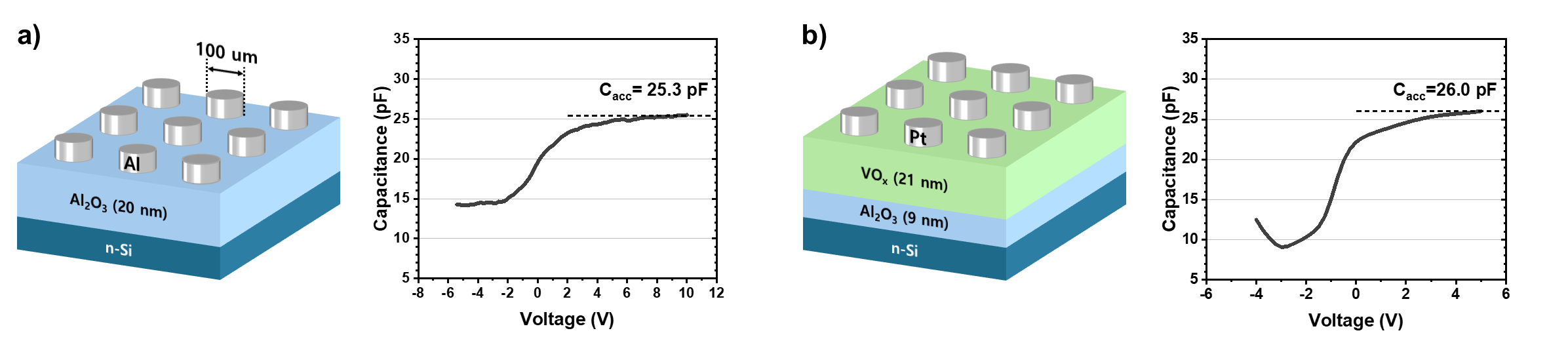
_

**Supporting Figure 6 | VO_x_ electrical dielectric constant (*ε*_r_) calculation through *C*–*V* measurements**. Schematic illustration of device structure (left) and *C*–*V* curves (right) of (a) Al/Al_2_O_3_/n-Si device, (b) Pt/VO_x_/Al_2_O_3_/n-Si device. The electrical dielectric constant (*ε*_r_) of VO_x_ layer was calculated using metal-oxide-semiconductor capacitor (MOSCAP) devices with a 100 μm diameter, based on the equation *ε*_r_=*C*_acc_·*d*/*ε*_0_·*A*. The accumulation capacitance (*C*_acc_)​ was measured from the *C*–*V* characteristics at a frequency of 1 MHz, and the exact thickness of VO_x_ layer was determined by AFM. An additional Al_2_O_3_ blocking layer was introduced to mitigate measurement errors caused by the high leakage current of the VO_x_ layer. This Al_2_O_3_ layer effectively reduced the leakage current, enabling reliable capacitance measurements by reducing the dissipation factor to an acceptable level. The experimentally calculated *ε*_r_ value of the single Al_2_O_3_ layer was approximately 7.28. Using this value, the *ε*_r_ value of the VO_x_ layer was subsequently calculated to be approximately 14.6.


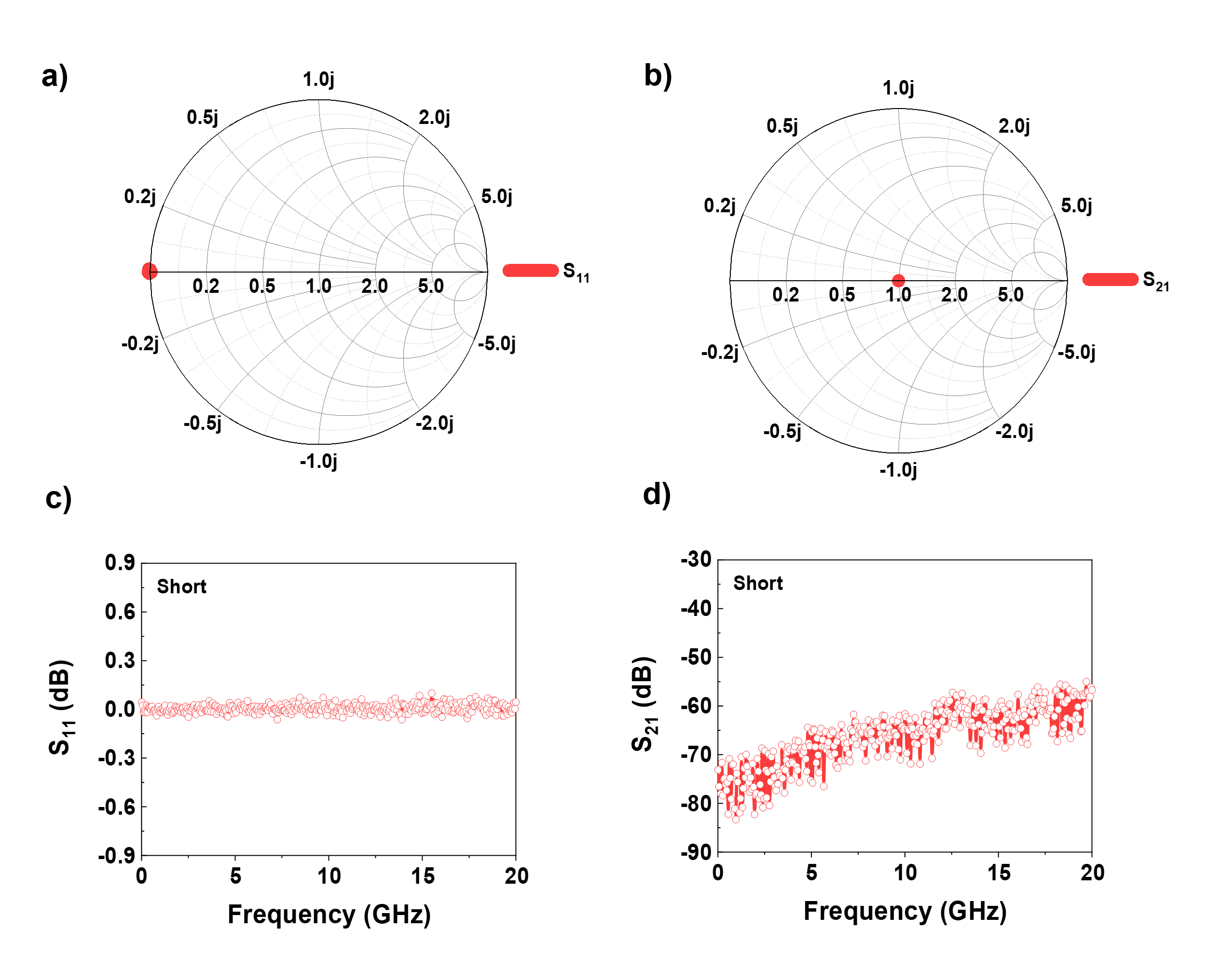


**Supporting Figure 7 | After-calibrated S_11_ and S_21_ results of a Short standard.** (a, b) Smith chart of the measured S_11_ and S_21_ responses for the Short standard, and (c, d) corresponding magnitude plots of the measured S_11_ and S_21_ parameters.


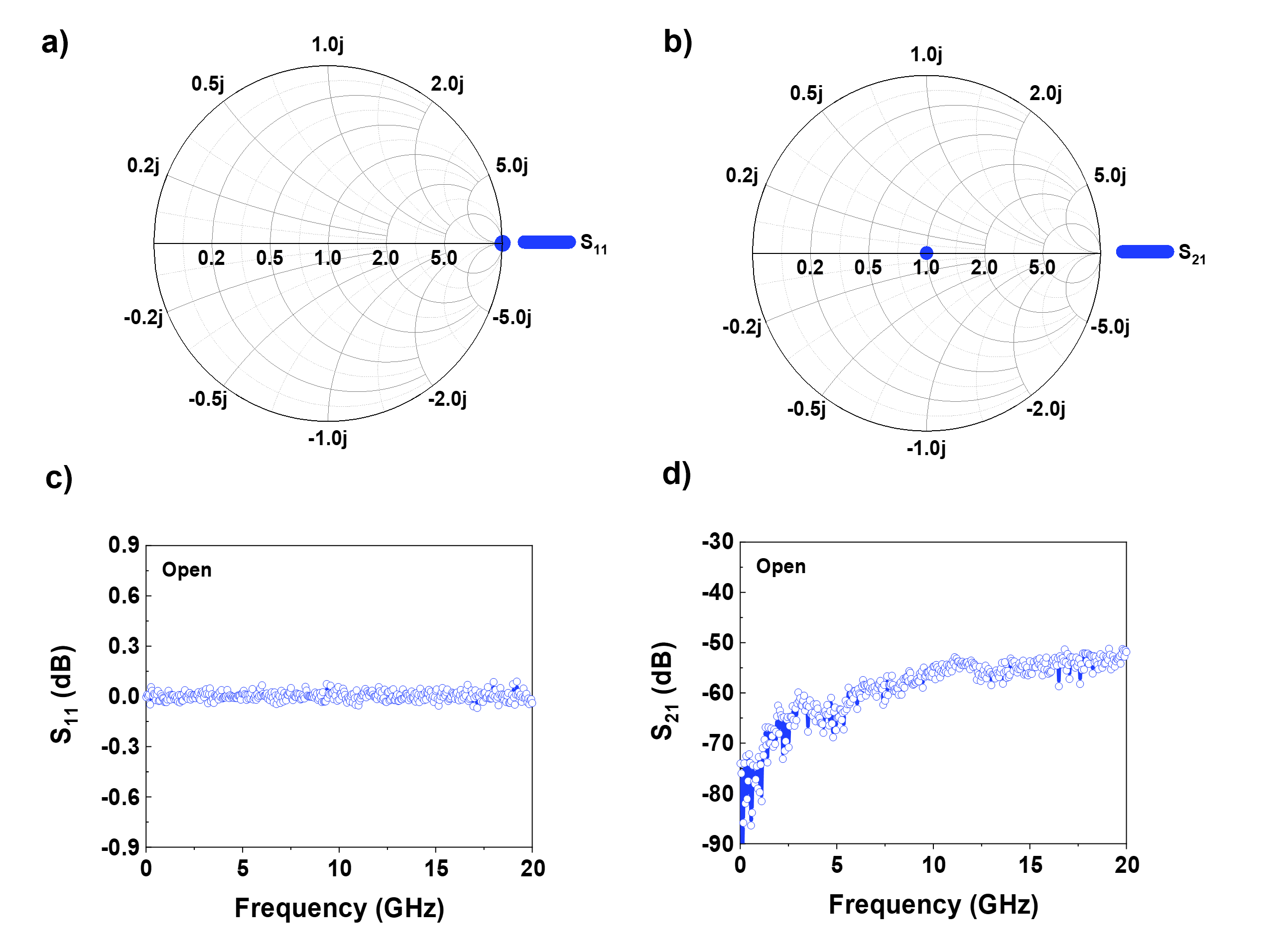


**Supporting Figure 8 | After-calibrated S_11_ and S_21_ results of an Open standard.** (a, b) Smith chart of the measured S_11_ and S_21_ responses for the Open standard, and (c, d) corresponding magnitude plots of the measured S_11_ and S_21_ parameters.


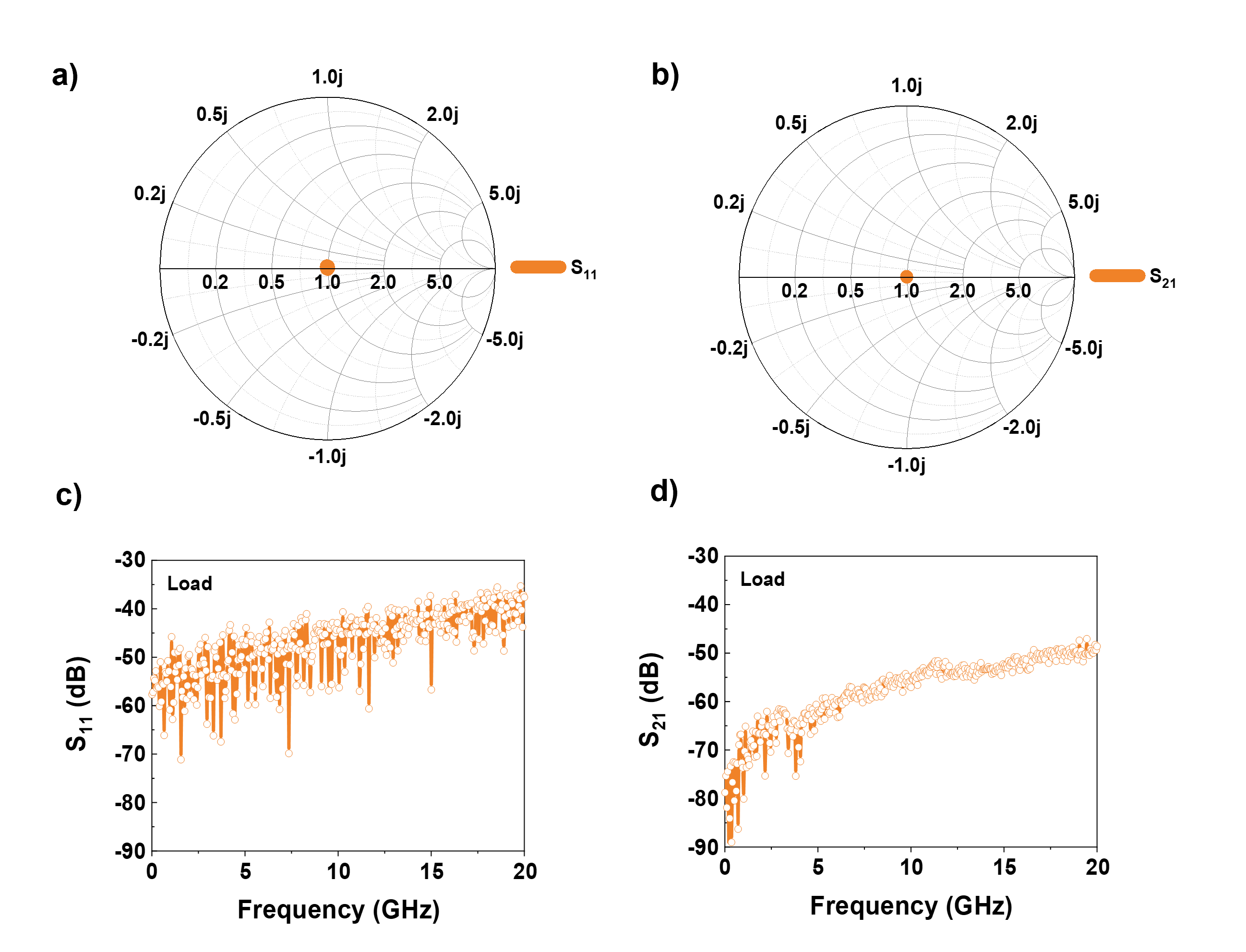


**Supporting Figure 9 | After-calibrated S_11_ and S_21_ results of a Load standard.** (a, b) Smith chart of the measured S_11_ and S_21_ responses for the Load standard, and (c, d) corresponding magnitude plots of the measured S_11_ and S_21_ parameters. The Load impedance is close to the 50 Ω characteristic impedance.


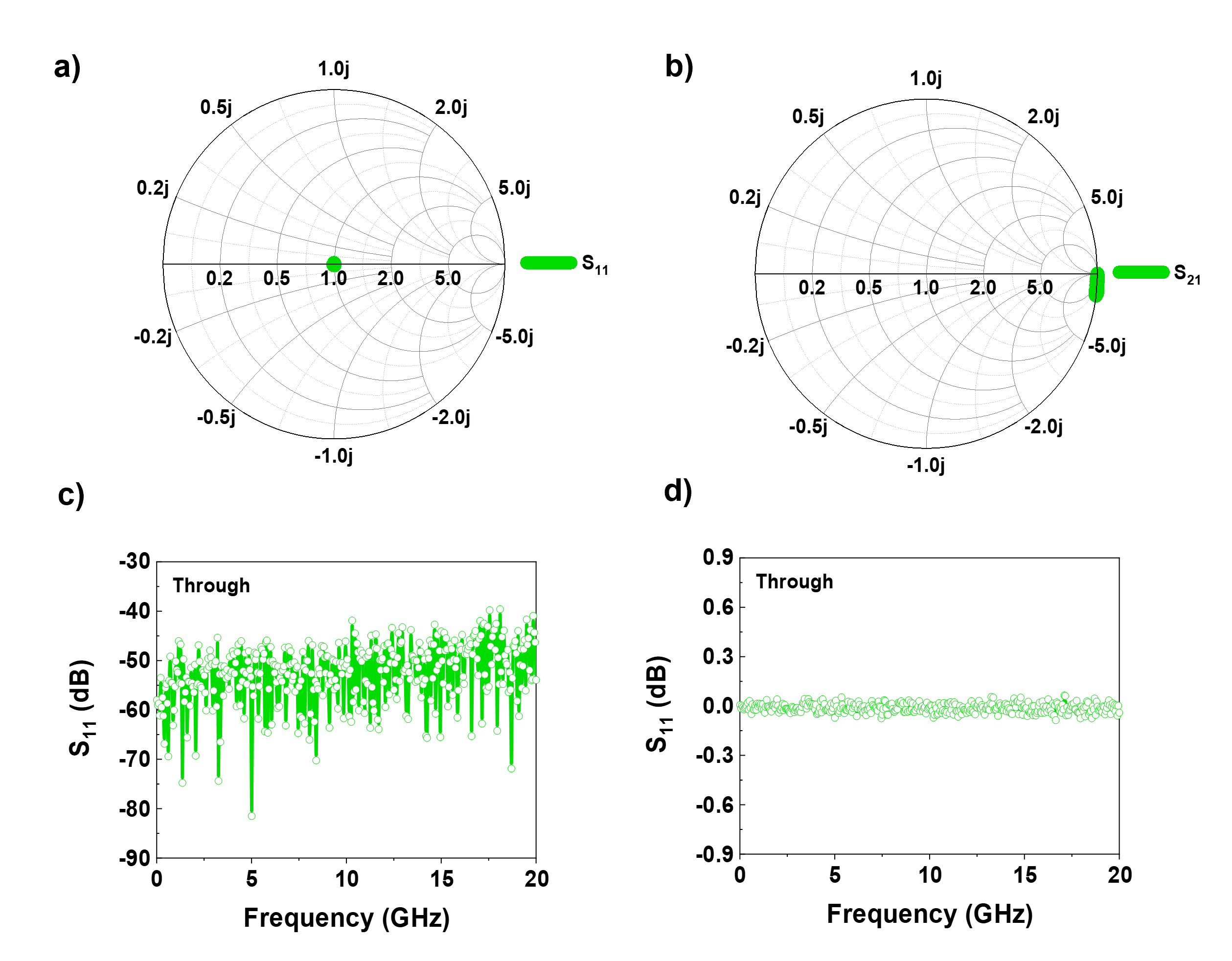


**Supporting Figure 10 | After-calibrated S_11_ and S_21_ results of a Through standard.** (a, b) Smith chart of the measured S_11_ and S_21_ responses for the Through standard, and (c, d) corresponding magnitude plots of the measured S_11_ and S_21_ parameters. The S_11_ return loss of the Through standard is more than 40 dB, and the S_21_ insertion loss of the standard is less than 0.08 dB.

**
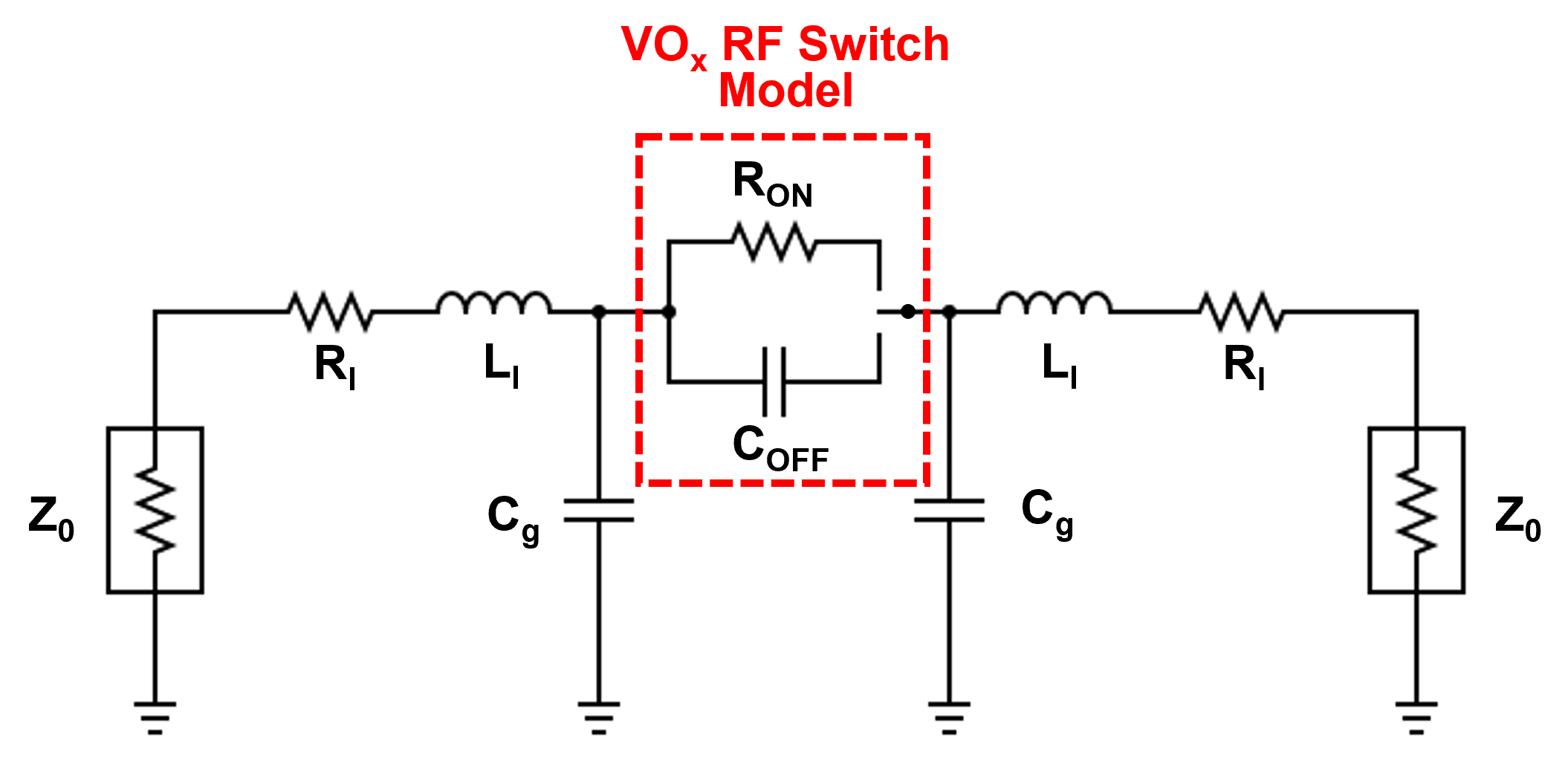
**

**Supporting Figure 11 | Lumped element equivalent circuit model of RF switch.** The schematic represents the equivalent circuit model of the VO_x_ RF switch. This model configuration includes the characteristic impedance (*Z*_0_​), a line resistor (*R*_l_​), a line inductor (*L*_l_), a shunt coupling capacitor to ground (*C*_g_), and either the switch OFF state capacitance (*C*_OFF_​) or the ON state resistance (*R*_ON_). The RLC components within the model, which represent interconnect parasitics, are de-embedded by on-chip Through and Open calibration structures.

**
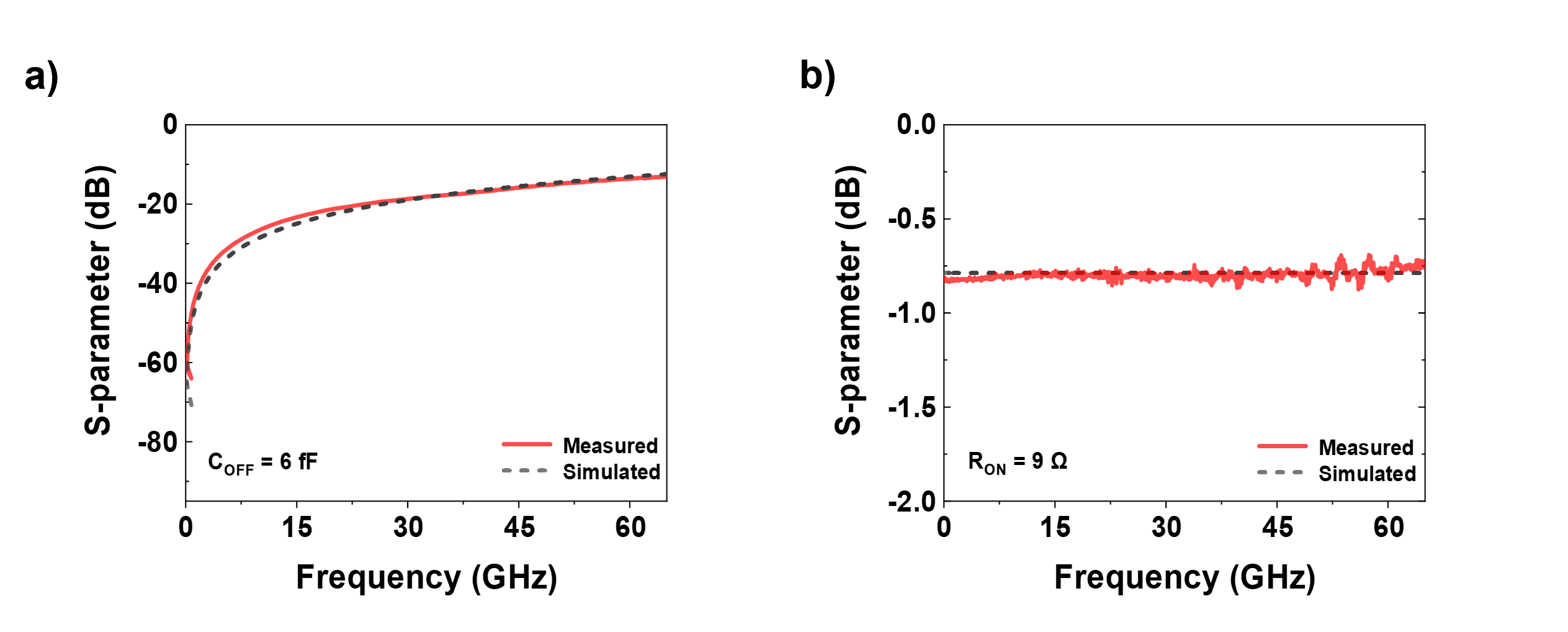
**

**Supporting Figure 12 | Performance evaluation of Ag/VO_x_/Au RF switches in the 67 GHz frequency range.** Radio-frequency characterization of the (a) OFF state and (b) ON state Ag/VO_x_/Au structured RF switches measured up to 67 GHz. The VO_x_ RF switch was measured with a current compliance of 1 mA. The values of *C*_OFF_​ (6 *f*F) and *R*_ON_​ (9 Ω) were extracted using a lumped element equivalent circuit model. Calculated from this, the cutoff frequency is 3 THz. The isolation at 67 GHz in the OFF state was 12.984 dB, and the insertion loss at 67 GHz in the ON state was 0.739 dB. The dashed lines are the simulated data from the equivalent circuit model.


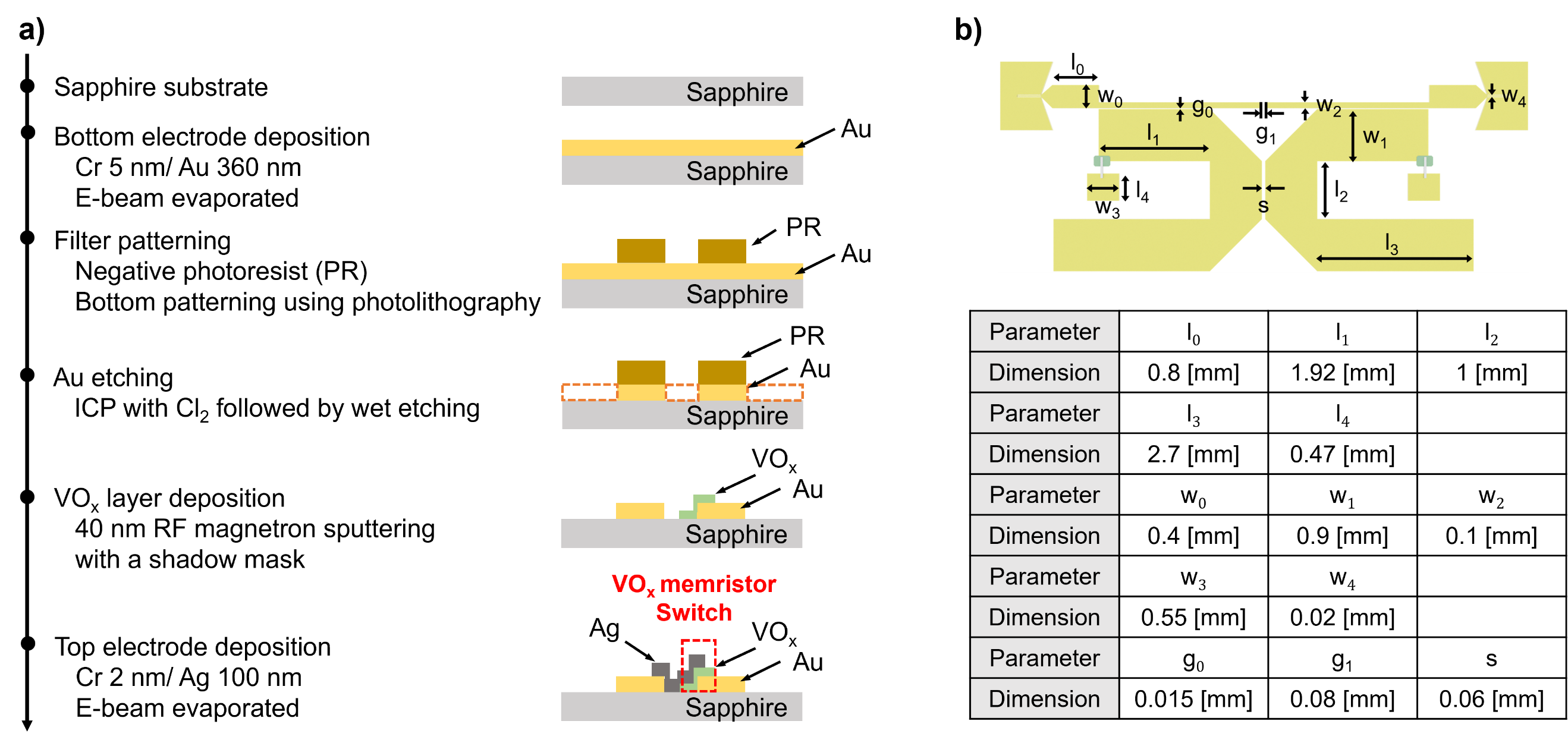


**Supporting Figure 13 | Fabrication process flow and component dimensions of the reconfigurable X-band Filter.** (a) Key fabrication process flow for filter with a side-view illustration. The red dashed box indicates the VO_x_ memristor switch. (b) Dimensions of each component of the reconfigurable X-band filter. The filter consists of two resonators, where the total resonator length (*l*_1_ *+ l*_2_ *+ l*_3_) is 5.62 mm. The coupling length (*l*_2_) is 1 mm, and the gap between the feed lines and the resonators (*g*_0_) is 0.015 mm. The final dimensions of the filter are as follows: *l*_0_=0.8, *l*_1_=1.92, *l*_2_=1, *l*_3_=2.7, *l*_4_=0.47, *w*_0_=0.4, *w*_1_=0.9, *w*_2_=0.1, *w*_3_=0.55, *w*_4_=0.02, *g*_0_=0.015, *g*_1_=0.08, *s*=0.06 (all in mm).

**
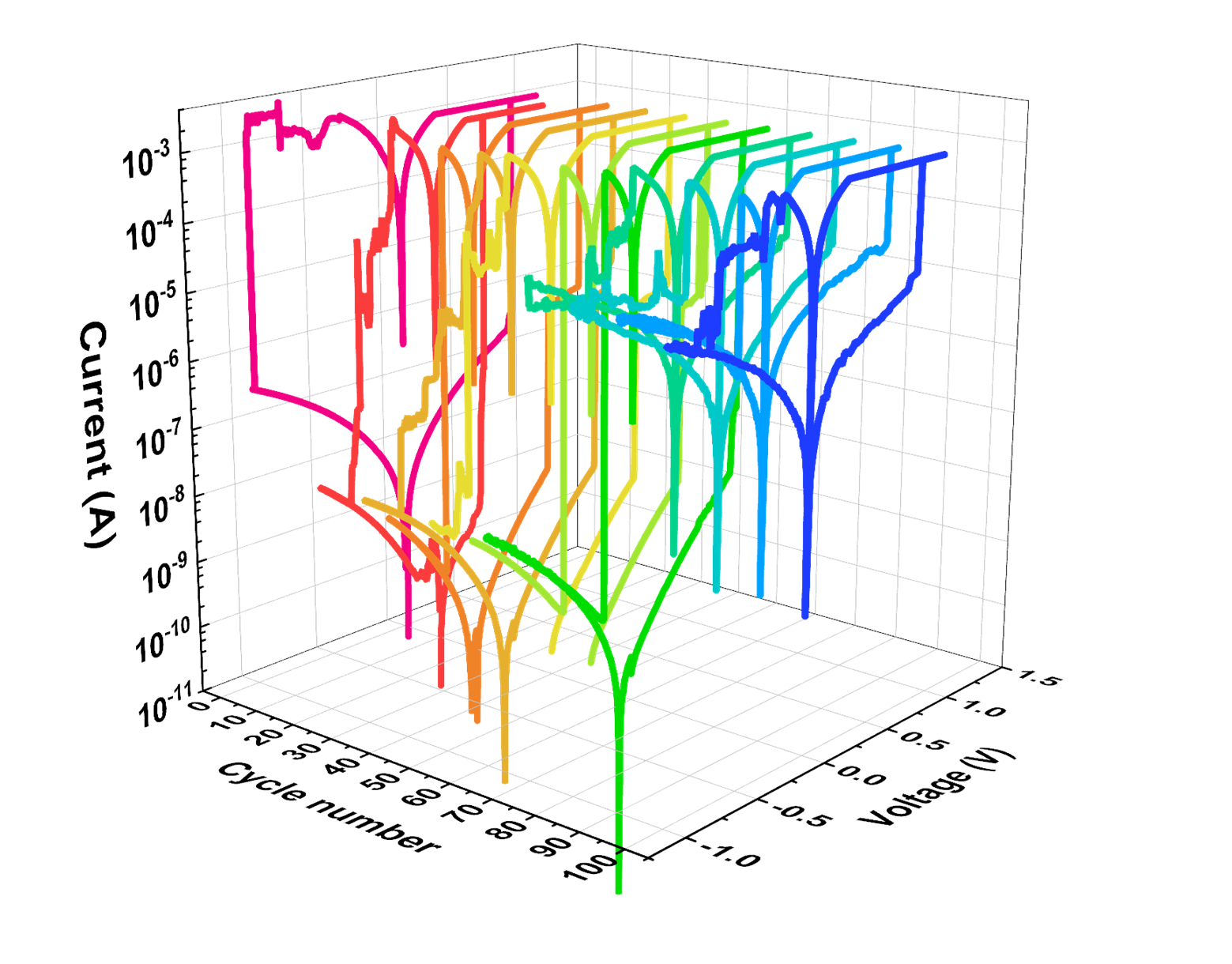
**

**Supporting Figure 14 | Typical DC cycling of VO_x_-based non-volatile resistive switches integrated within a filter structure.** The reliability of the VO_x_ device integrated into the filter was tested under DC voltage sweeps with a SET compliance current of 1 mA, demonstrating consistent switching behavior with an endurance of over 100 cycles. Additionally, the device exhibited low voltage operation characteristics, with the *V*_SET_ and *V*_RESET_ remaining within ±1 V, highlighting its suitability for energy-efficient and low-power applications.

**
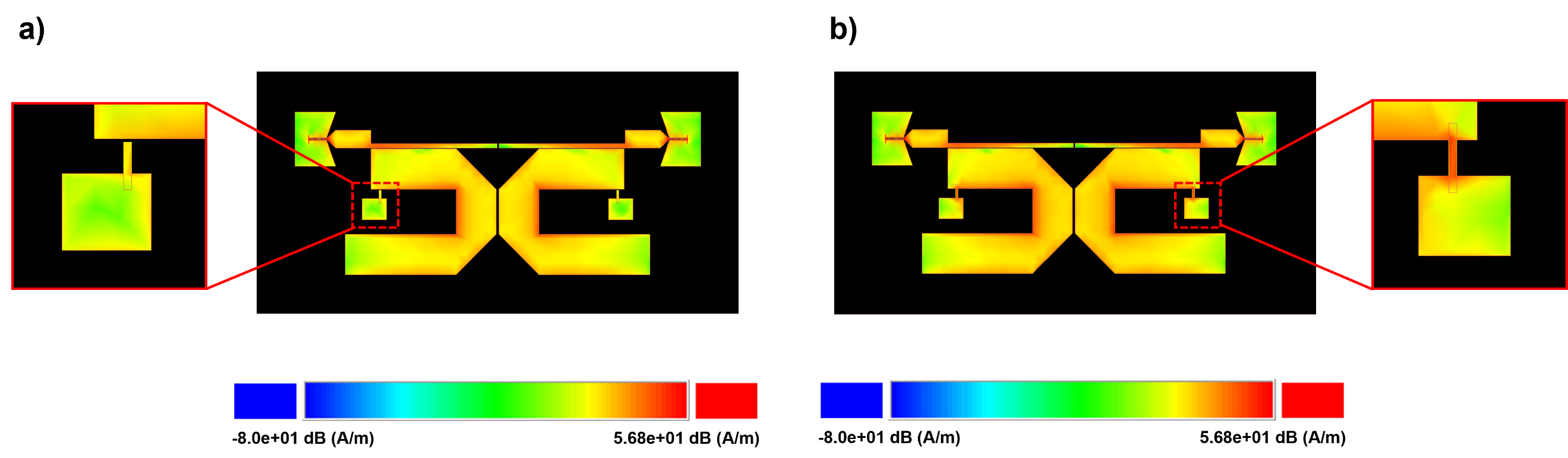
**

**Supporting Figure 15 | Simulated current density distribution of the filter model.** (a) shows the current density distribution of the filter when the main patch and the parasitic patch are not connected, whereas (b) represents the current density distribution when the main patch and the parasitic patch are connected via a metal transmission line. In both configurations, the current distribution is symmetrical around the y-axis. The current density analysis reveals that the connection between the main and parasitic patches results in a significant increase in current density within the parasitic patch, as indicated by the shift toward red in the color gradient.

**Supporting Note**

**Supporting Note 1 | Detailed results and discussion of XPS analysis.**

This section provides detailed XPS analysis results **(Figure 1d** in the main text), including peak fitting processes, additional spectral data, and comprehensive discussions on the chemical states and oxygen vacancy-related defects in the VO_x_ layer. These findings complement the main text by offering in-depth insights into the material's composition and properties.

1. **XPS Peak fitting condition**

The XPS spectra present V 2p and O 1s core level peaks acquired from the 10 nm-etched surface of VO_x_ layer. Because the spectra were obtained from the etched surface, conventional binding energy calibration using the adventitious carbon peak could not be applied to these spectra. The background spectra were fitted using a Shirley-type background, and a mixed Lorentzian-Gaussian curve (ratio corresponding to 70% Gaussian and 30% Lorentzian) was applied to fit each individual peak.^[1]^ The area ratio between V 2p_3/2_ and V 2p_1/2_ energy levels was fixed to a 2:1 due to the spin-orbit coupling splitting of the V 2p energy level, and the peak difference of V 2p_1/2_ and V 2p_3/2_ of each oxidation state was constrained with spin-orbit splitting difference value of V 2p spectrum.^[2-4]^ These values are reported to be in the range of 7-8 eV which can be found in many literature sources and the NIST Database.^[3,5-6]^

1. **Satellite peak**

Overall spectra were deconvoluted by including a few satellite peaks (dark grey lined). The one which is located in the binding energy of 525.2 eV is due to the strong hybridization of V 3d and O 2p energy levels.^[5,7-8]^ Additionally, other satellite peaks at 519.6 and 516.9 eV are observed in between V 2p_1/2_ and V 2p_3/2_ spectra and these satellite peaks can be elucidated by X-ray satellite which comes from O 1s core level.^[9-10]^

1. **Detailed discussions on the chemical states in the VO_x_ layer**

The XPS analysis in the main text inferred that the VO_x_ film is oxygen-deficient, a conclusion supported by additional explanations and evidence provided here. Among various VO_x_ phases, the V₂O₅ phase is the most thermodynamically stable under normal temperature and pressure conditions, as evidenced by the Ellingham diagram showing the Gibbs free energy of formation for VO_x_ phases and the phase stability diagram of VO_x_.^[11-12]^ Therefore, the VO_x_ layer in this study is considered to be oxygen-deficient from the stable V_2_O_5_. Generally, the presence of numerous oxygen vacancies in the metal-oxide layer facilitates Ag^+^ ion migration for filament formation at low voltage by reducing the activation energy of Ag^+^ diffusion and giving the diffusion pathways through oxygen vacancies compared to that through the stoichiometric metal-oxide. For example, the significant facilitation of Ag^+^ diffusion in an amorphous SiO_2_ switching layer with oxygen vacancy is evidenced by NEB simulations, which reveal a remarkably low diffusion activation energy of 0.29 eV for Ag^+^ ions interacting with oxygen vacancies. This low activation energy highlights the critical role of oxygen vacancies in reducing diffusion barriers, making them a key factor in enhancing ion mobility compared to stoichiometric oxides, as reported by Patel et al.^[13]^ Furthermore, according to the DFT calculations to examine the role of oxygen vacancies in facilitating the diffusion of Ag^+^, oxygen vacancies act as a reduction site, providing efficient pathways for Ag^+^ migration within the resistive switching layer.^[14]^ This interaction not only stabilizes Ag^+^ locally but also supports the formation of conductive filaments, ultimately enhancing the performance and uniformity of VO_x_ devices. In this context, the fast resistive switching at low voltage of the device in this study can be elucidated by facilitated Ag^+^ diffusion thanks to this oxygen-vacancy-mediate diffusion pathway.

**Supporting Note 2 | ADS simulation.**

The X-band BPF presented in this work was optimized using a full-wave electromagnetic (EM) simulation approach implemented in the Momentum simulator of ADS. The simulation was based on the actual physical layout of the fabricated filter.

To address discrepancies between idealized circuit responses and real-world effects due to material properties and fabrication variations, we applied a two-fold parameter adjustment strategy. First, the dielectric properties of the substrate were carefully refined. Specifically, the relative permittivity (*ε*_r_) and the loss tangent (tan *δ*) in the EM model were adjusted to reflect the dispersion characteristics and tolerances of the fabricated substrate.

Second, we modeled finite conductor losses, which become significant due to the use of thin films of noble metals such as Ag or Au. While ADS provides default bulk conductivity values (e.g., *σ*_Au_ ≈ 4.1 × 10^7^ S/m), real thin-film implementations exhibit lower effective conductivities because of factors such as surface roughness, grain boundary scattering, and skin effect at high frequencies. Based on the fabrication and material considerations reported by S. Courréges et al.^[15]^, we adjusted the conductivity parameters to reflect more realistic conditions encountered in thin-film implementations, thereby achieving improved agreement between the simulated and experimentally observed insertion loss.

To further enhance simulation accuracy, the mesh density was locally refined in areas sensitive to field concentration, including discontinuities, coupling gaps, and resonator edges. This step minimized numerical artifacts and ensured convergence of the calculated S-parameters, which is particularly important when analyzing narrowband filters in the gigahertz range where field distribution plays a critical role. Overall, this multi-parameter optimization process enabled realistic modeling of the filter's RF performance and allowed us to closely match the expected behavior of the fabricated structure.

**Supporting Tables**

**Supporting Table 1 | Non-volatile resistive switching performance comparison of VO_x_-based memristor.**

| Device type | Structure | Oxide  thickness | V_SET_ / V_RESET_ | ON/OFF ratio | Cycling endurance | Retention | Program  time | Overlap area | Mechanism | REF |
| --- | --- | --- | --- | --- | --- | --- | --- | --- | --- | --- |
| ReRAM | Ti/VO_x_/Pt | 100 [nm] | 1 [V]  −1.1 [V] | ~92 | 10^4^ | > 10^4^ [s]  (360 [K]) | - | - | Oxygen vacancy | [16] |
|  | Pt/Ti/VO_x_/Pt | 100 [nm] | 2.7 [V]  −1.7 [V] | 2 | 200 | 10^3^ [s]  (300 [K]) | - | 300 [µm] diameter | Interfacial redox | [17] |
|  | Pt/VO_x_/Pt | 148.9 [nm] | 1.93 [V]  −0.6 [V] | 10 | 350 | 5 × 10^3^ [s]  (300 [K]) | 28.5 [ms] | 300 [µm] diameter | Metal-insulator transition and oxygen vacancy | [18] |
|  | Au/VO_x_/Indium Tin Oxide | Nanosheets | −0.42 [V]  2.26 [V] | > 50 | 120 | 1.5 × 10^3^ [s]  (300 [K]) | - | 100 [µm] diameter | Oxygen vacancy | [19] |
| CBRAM | Ag/VO_x_/Pt | 35 [nm] | 0.23 [V]  −0.07 [V] | > 10^2^ | > 8 × 10^3^ | > 4 × 10^4^ [s]  (300 [K]) | 100 [ns] | 100 [µm] diameter | Silver ion | [20] |
|  | Ag/VO_x_/Au | 40 [nm] | 2.5 [V]  −0.9 [V] | > 10^7^ | ~10^3^ | > 2 × 10^5^ [s]  (300 [K]) | 100 [ns] | 4 [µm] × 4 [µm] | Silver ion | This work |

**Supporting Table 2 | RF switch technologies comparison table.**

| Device type | Structure | Nonvolatility | Switching voltage | Switching time | Switching ratio | Maximum operating frequency | Insertion loss | Isolation | Cutoff frequency | Operation environment | REF |
| --- | --- | --- | --- | --- | --- | --- | --- | --- | --- | --- | --- |
| MEMS | Au-Ru contact array | No | 47 - 60 [V] | 2.2 [µs] | - | 40 [GHz] | 1.0 [dB] | 20 [dB] | 3.8 [THz] | Hermetic packaging | [21] |
|  | Metal contact | No | 80 - 90 [V] | 5 [µs] | - | 40 [GHz] | 0.5 [dB] | 20 [dB] | 12.4 [THz] | Hermetic packaging | [22] |
| PCM | Cr/GeTe/Cr | Yes | 1 - 4 [V] | 0.5 - 20 [µs] | - | 25 [GHz] | 1.0 [dB] | 20 [dB] | 6 [THz] | Heater needed | [23] |
|  | AlCu/GeTe/Si | Yes | 1.1 [V] | < 120 [ns] | 10^5^ | 40 [GHz] | 0.15 [dB] | 14 [dB] | 21 [THz] | Heater needed | [24] |
| 2D-based RF switch | Au/hexagonal-boron nitride/Au | Yes | 1.5 [V] | 15 [ns] | 10^5^ | 220 [GHz] | 0.5 [dB] | 10 [dB] | 129 [THz] | Ambient condition | [25] |
|  | Au/MoS_2_/Au | Yes | 1.3 [V] | 30 [ns] | 10^3^ | 50 [GHz] | 0.3 [dB] | 15 [dB] | 100 [THz] | Ambient condition | [26] |
| Oxide-based RF switch | Ni/HfO_2_/Ni | Yes | 0.5 - 1 [V] | 10 [ns] | 10^4^ | 110 [GHz] | 2 [dB] | 13 [dB] | 0.84 [THz] | Ambient condition | [27] |
|  | Pt/TiO_2-x_/ Pt | Yes | ~2 [V] | - | - | 10 [GHz] | 2 [dB] | 32 [dB] | 2.8 [THz] | Ambient condition | [28] |
|  | Ag/VO_x_/Au | Yes | 2.5 [V] | 100 [ns] | 10^7^ | 67 [GHz] | 0.45 [dB] | 20 [dB] | 4.5 [THz] | Ambient condition | This work |

**Supporting Table 3 | Performance comparison of the X-band reconfigurable filter.**

| Tuning components | FBW_3-dB_ | Insertion loss | Range | Quality factor | Bias voltage | Size | Tuning speed | Static power consumption | REF |
| --- | --- | --- | --- | --- | --- | --- | --- | --- | --- |
| PIN diode | 54, 31.15 [%] | 2.02, 1.127 [dB] | 7.6 - 8.3 [GHz] | 15.4, 24.5 | - | 14 × 14 [mm^2^] | - | - | [29] |
| Barium strontium titanate capacitor | 15 [%] | 2.2 - 2.7 [dB] | 10 - 10.56 [GHz] | < 73 | 30 [V] | 3.1 × 6.9 [mm^2^] | - | - | [30] |
| MEMS switch | 5.8 [%] | 2.6 - 2.9 [dB] | 12 - 15 [GHz] | 213.7 | 60 [V] | 5 × 4 [mm^2^] | 6 - 8 [µs] | Near 0 | [31] |
| VO_2_ switch | 12, 13 [%] | 5 [dB] | 8.6 - 9.2 [GHz] | 71.6, 70.7 | 60 [V] | 9 × 7 [mm^2^] | - | 1.8 [W] (pulse) | [32] |
| GeTe switch | 6.4, 6.5 [%] | 3.2, 2.6 [dB] | 7.45 - 8.07 [GHz] | 116.4, 124.1 | 15 to 20 [V] | 3.9 × 10.8 [mm^2^] | 6 [µs] | 0.5~1.5 [W] | [33] |
| VO_x_ switch | 8.5, 7.3 [%] | 9.6, 5.6 [dB] | 7.6 - 8.2 [GHz] | 89.4, 112.3 | 0 [V] | 3.9 × 10.8 [mm^2^] | 100 [ns] | Near 0 | This work |

**Supporting References**

[1] D. A. Shirley, *Physical Review B* **1972**, *5* (12), 4709, https://doi.org/10.1103/PhysRevB.5.4709.

[2] J. F. S. Moulder, W.F.; Sobol, P.E.; Bomben, K.D., *Handbook of X-ray Photoelectron Spectroscopy*, Perkin-Elmer Corp, Eden Prairie, MN **1992**.

[3] C. D. N. Wagner, A.V.; Kraut-Vass, A.; Allison, J.W.; Powell, C.J.; Rumble, J.R., Jr., (Preprint) v3.4, submitted.

[4] G. Silversmit, D. Depla, H. Poelman, G. B. Marin, R. De Gryse, *Journal of Electron Spectroscopy and Related Phenomena* **2004**, *135* (2-3), 167, https://doi.org/10.1016/j.elspec.2004.03.004.

[5] R. Zimmermann, R. Claessen, F. Reinert, P. Steiner, S. Hüfner, *Journal of Physics Condensed Matter* **1998**, *10* (25), 5697, https://doi.org/10.1088/0953-8984/10/25/018.

[6] R. J. Colton, A. M. Guzman, J. W. Rabalais, *Journal of Applied Physics* **1978**, *49* (1), 409, https://doi.org/10.1063/1.324349.

[7] A. Rakshit, M. Mukherjee, S. Chakraborty, *Materials Science in Semiconductor Processing* **2018**, *88*, 127, https://doi.org/10.1016/j.mssp.2018.07.040.

[8] G. A. Sawatzky, D. Post, *Physical Review B* **1979**, *20* (4), 1546, https://doi.org/10.1103/PhysRevB.20.1546.

[9] H. T. T. Nguyen, D. Jung, C. Y. Park, D. J. Kang, *Materials Chemistry and Physics* **2015**, *165*, 19, https://doi.org/10.1016/j.matchemphys.2015.05.053.

[10] M. Shahid, J. Liu, Z. Ali, I. Shakir, M. F. Warsi, *Journal of Power Sources* **2013**, *230*, 277, https://doi.org/10.1016/j.jpowsour.2012.12.033.

[11] A. J. Bergerud, *Phase Stability and Transformations in Vanadium Oxide Nanocrystals*, University of California, Berkeley, **2016**.

[12] T. Shyrokykh, L. Neubert, O. Volkova, S. Sridhar, *Processes* **2023**, *11* (6), https://doi.org/10.3390/pr11061646.

[13] K. Patel, J. Cottom, M. Bosman, A. J. Kenyon, A. L. Shluger, *Microelectronics Reliability* **2019**, *98*, 144, https://doi.org/10.1016/j.microrel.2019.05.005.

[14] U. Rasheed, M. Imran, F. Hussain, U. Mumtaz, A. M. Tighezza, R. M. A. Khalil, M. F. Ehsan, *Journal of Physics and Chemistry of Solids* **2024**, *193*, https://doi.org/10.1016/j.jpcs.2024.112214.

[15] R. A. Matula, *Journal of Physical and Chemical Reference Data* **1979**, *8* (4), 1147, https://doi.org/10.1063/1.555614.

[16] W. Li, Y. Tuo, W. Mi, D. Wang, M. Wang, L. Zhou, J. Zhao, *Vacuum* **2023**, *209*, https://doi.org/10.1016/j.vacuum.2022.111794.

[17] S. Nirantar, E. Mayes, M. A. Rahman, T. Ahmed, M. Taha, M. Bhaskaran, S. Walia, S. Sriram, *Advanced Electronic Materials* **2019**, *5* (12), https://doi.org/10.1002/aelm.201900605.

[18] G. Li, J. Wei, H. Wang, R. Xiong, D. Wang, Y. Zhu, Y. Liu, Z. Zou, J. Xu, H. Ma, *Ceramics International* **2021**, *47* (19), 27479, https://doi.org/10.1016/j.ceramint.2021.06.171.

[19] M. K. Hota, D. H. Nagaraju, M. N. Hedhili, H. N. Alshareef, *Applied Physics Letters* **2015**, *107* (16), https://doi.org/10.1063/1.4933335.

[20] J. Ryu, K. Park, D. P. Sahu, T. S. Yoon, *ACS Appl Mater Interfaces* **2024**, *16* (20), 26450, https://doi.org/10.1021/acsami.4c04874.

[21] R. Stefanini, M. Chatras, P. Blondy, G. M. Rebeiz, *Journal of Microelectromechanical Systems* **2011**, *20* (6), 1324, https://doi.org/10.1109/JMEMS.2011.2170822.

[22] C. D. Patel, G. M. Rebeiz, *IEEE Transactions on Microwave Theory and Techniques* **2012**, *60* (10), 3096, https://doi.org/10.1109/TMTT.2012.2211888.

[23] Y. Shim, G. Hummel, M. Rais-Zadeh, in *Proceedings of the IEEE International Conference on Micro Electro Mechanical Systems (MEMS)* **2013**, 237-240.

[24] A. Leon, B. Reig, E. Perret, F. Podevin, D. Saint-Patrice, V. Puyal, J. Lugo-Alvarez, P. Ferrari, *IEEE Transactions on Microwave Theory and Techniques* **2020**, *68* (1), 60, https://doi.org/10.1109/TMTT.2019.2946145.

[25] M. Kim, E. Pallecchi, R. Ge, X. Wu, G. Ducournau, J. C. Lee, H. Happy, D. Akinwande, *Nature Electronics* **2020**, *3* (8), 479, https://doi.org/10.1038/s41928-020-0416-x.

[26] M. Kim, R. Ge, X. Wu, X. Lan, J. Tice, J. C. Lee, D. Akinwande, *Nature Communications* **2018**, *9* (1), https://doi.org/10.1038/s41467-018-04934-x.

[27] S. C. Chen, Y. T. Yang, Y. C. Tseng, K. D. Chiou, P. W. Huang, J. H. Chih, H. Y. Liu, T. T. Chou, Y. Y. Jhang, C. W. Chen, C. H. Kuan, E. M. Ho, C. H. Chien, C. N. Kuo, Y. T. Cheng, D. H. Lien, *ACS Nano* **2024**, https://doi.org/10.1021/acsnano.4c11846.

[28] B. e. a. Ruxandra, in *Proc. Innov. Sustainability Conf.* **2017**, 41–42.

[29] M. H. Masood, S. B. Suseela, *The Journal of Engineering* **2018**, *2018* (3), 162.

[30] S. Courréges, Y. Li, Z. Zhao, K. Choi, A. T. Hunt, J. Papapolymerou, *IEEE Transactions on Microwave Theory and Techniques* **2009**, *57* (12), 2872, https://doi.org/10.1109/TMTT.2009.2034046.

[31] A. Pothier, J. C. Orlianges, G. Zheng, C. Champeaux, A. Catherinot, D. Cros, P. Blondy, J. Papapolymerou, in *IEEE Transactions on Microwave Theory and Techniques* **2005**, 354-360.

[32] D. Bouyge, A. Crunteanu, J. C. Orlianges, D. Passerieux, C. Champeaux, A. Catherinot, A. Velez, J. Bonache, F. Martin, P. Blondy, in *APMC 2009 - Asia Pacific Microwave Conference 2009* **2009**, 2332-2335.

[33] M. Wang, F. Lin, M. Rais-Zadeh, in *SiRF 2016 - 2016 IEEE 16th Topical Meeting on Silicon Monolithic Integrated Circuits in RF Systems* **2016**, 38-41.
